# E4BP4 Coordinates Circadian Control of Cognition in Delirium

**DOI:** 10.1002/advs.202200559

**Published:** 2022-06-17

**Authors:** Min Chen, Li Zhang, Mingting Shao, Jianhao Du, Yifei Xiao, Fugui Zhang, Tianpeng Zhang, Yifang Li, Qianqian Zhou, Kaisheng Liu, Zhigang Wang, Baojian Wu

**Affiliations:** ^1^ Institute of Molecular Rhythm and Metabolism Guangzhou University of Chinese Medicine Guangzhou 510006 China; ^2^ College of Pharmacy Jinan University Guangzhou 510632 China; ^3^ Guangdong‐Hongkong‐Macau Institute of CNS Regeneration Jinan University Guangzhou 510632 China; ^4^ Shenzhen People's Hospital (The Second Clinical Medical College Jinan University; The First Affiliated Hospital Southern University of Science and Technology) Shenzhen 518119 China; ^5^ Department of Intensive Care Unit First Affiliated Hospital of Jinan University Guangzhou 510630 China

**Keywords:** circadian rhythm, delirium, E4BP4, ERK1/2, microglia

## Abstract

Improved understanding of the etiologies of delirium, a common and severe neuropsychiatric syndrome, would facilitate the disease prevention and treatment. Here, the authors invesitgate the role of circadian rhythms in the pathogenesis of delirium. They observe perturbance of circadian rhythms in mouse models of delirium and disrupted clock gene expression in patients with delirium. In turn, physiological and genetic circadian disruptions sensitize mice to delirium with aggravated cognitive impairment. Likewise, global deletion of *E4bp4* (E4 promoter‐binding protein), a clock gene markedly altered in delirium conditions, results in exacerbated delirium‐associated cognitive decline. Cognitive decline in delirium models is attributed to microglial activation and impaired long‐term potentiation in the hippocampus. Single‐cell RNA‐sequencing reveals microglia as the regulatory target of *E4bp4*. *E4bp4* restrains microglial activation via inhibiting the ERK1/2 signaling pathway. Supporting this, mice lacking in microglial *E4bp4* are delirious prone, whereas mice with *E4bp4* specifically deleted in hippocampal CA1 neurons have a normal phenotype. Mechanistically, *E4bp4* inhibits ERK1/2 signaling by trans‐repressing *Mapk1/3* (genes encoding ERK1/2) via direct binding to a D‐box element in the promoter region. These findings define a causal role of clock dysfunction in delirium development and indicate *E4bp4* as a regulator of cognition at the crosstalk between circadian clock and delirium.

## Graphical abstract

In this study, circadian disruption sensitizes mice to delirium with aggravated cognitive impairment due to down‐regulation of hippocampal E4BP4. Mechanistically, E4BP4 promotes hippocampal long‐term potentiation and cognitive performance through restraining ERK1/2 cascade and microglial activation. These findings indicate E4BP4 as a regulator of cognition at the crosstalk between circadian clock and delirium and as a therapeutic target for delirium.

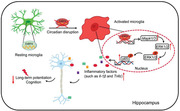

## 1 Introduction

Delirium is a severe neuropsychiatric syndrome with key features of acute cognitive and attentional deficits, affecting about 20% of patients in general hospital setting.^[^ ^1^ ^]^ The prevalence can be as high as 60–80% for critically ill patients.^[^ ^2^ ^]^ Delirium is associated with prolonged hospitalization and increased morbidity (e.g., long‐term cognitive impairment and worsened motor‐sensory function) and mortality, and thus is a major health care concern.^[^ ^3^ ^]^ The cost of managing delirium is estimated to be 164 billion dollars annually in the US.^[^ ^4^, ^5^ ^]^ A number of risk factors have been described for delirium, including acute medical illness (e.g., sepsis and liver failure), trauma (e.g., fractures and severe burns), surgery and medications (e.g., benzodiazepines, antihistamines, and opioids).^[^ ^3^ ^]^ Given that the etiologies of delirium are diverse and multifactorial, there are likely multiple neurobiological mechanisms underlying delirium pathogenesis, including neuroinflammation, altered brain energy metabolism, brain vascular dysfunction, neurotransmitter disturbance, and weakened neural network connectivity.^[^ ^6^ ^]^ Unfortunately, drug treatments for delirium (such as cholinesterase inhibitors and antipsychotics) are largely ineffective, suggesting inadequate understanding of its pathophysiology.^[^ ^6^, ^7^ ^]^ The current goal of delirium treatment is to manage the precipitating factors and militate the complications.^[^ ^6^ ^]^ Therefore, it is of great clinical value to unravel mechanistic routes that cause delirium and to identify plausible targets for therapeutic interventions.

Almost all organisms in the planet are subject to circadian rhythms which allow them to anticipate and adapt to the changing environment (e.g., the light‐dark cycle and food availability).^[^ ^8^ ^]^ In mammals, circadian rhythms are maintained and regulated by the clock system consisting of the master clock in the suprachiasmatic nucleus (SCN) of hypothalamus and the clocks in peripheral tissues such as liver, lung, and skeletal muscle.^[^ ^9^, ^10^ ^]^ The core of mammalian clock is a negative feedback loop composed of several transcription factors, namely, CLOCK (circadian locomotor output cycles kaput), BMAL1 (brain and muscle ARNT‐like 1), PERs (periods), and CRYs (cryptochromes).^[^ ^11^ ^]^ CLOCK forms a dimer with BMAL1 to stimulate the transcription of *Pers*, *Crys*, and many other genes.^[^ ^12^ ^]^ After the transcripts of *Pers* and *Crys* are translated in the cytoplasm, their proteins move into the nucleus to inhibit the activity of BMAL1/CLOCK and stop more transcripts of *Pers* and *Crys* being made.^[^ ^13^ ^]^ As PER and CRY proteins degrade, CLOCK and BMAL1 are freed to promote Per and *Cry* transcription again and a new cycle starts.^[^ ^14^ ^]^ BMAL1/CLOCK also promotes the expression of REV‐REBs and RORs, which controls *Bmal1* transcription, thus acting as an auxiliary loop to reinforce the core clock and to help maintain its robust oscillation.^[^ ^15^ ^]^

E4BP4 (E4 promoter‐binding protein 4, also known as NFIL3) is a member of the basic leucine zipper (bZIP) family of transcription factors.^[^ ^16^ ^]^ E4BP4 functions to repress the transcription of target genes by competing for D‐box binding with the proline‐alanine rich (PAR) subfamily of bZIP transcription factors (functioning as transcriptional activators), and recruiting HDAC2 (histone deacetylase 2) and G9a (a histone methyltransferase) via a repression domain composed of amino acids 299–363 near the C‐terminus.^[^ ^17^, ^18^ ^]^ The role of E4BP4 in immune system has been well‐recognized. It is critical for development of immune cells such as NK, Th17, and CD8*α* ^+^ dendritic cells, and is involved in macrophage activation, IgE class switching, and polarization of T cell responses.^[^ ^19^, ^20^ ^]^ E4BP4 is also implicated in regulation of circadian rhythms via repressing the expression of PERs and may be regarded as a component of molecular clock.^[^ ^17^ ^]^ In addition, E4BP4 has an important role in osteoblast function, heart failure, ovulation, and cell metabolism.^[^ ^21^ ^]^ However, the E4BP4's role in the hippocampus and its relevance to cognitive function are largely unknown.

Current literature suggests a relationship between delirium and circadian rhythms and a potential relevance of circadian rhythms in delirium pathogenesis.^[^ ^22^ ^]^ Patients with delirium are associated with marked disruptions of circadian rhythms as evidenced by sleep–wake cycle disturbance, melatonin and cortisol arrhythmicity, as well as altered clock gene expression.^[^ ^22^, ^23^, ^24^, ^25^, ^26^ ^]^ Environmental factors (e.g., constant lighting and noise) specific to the intensive care unit (ICU) and highly related to circadian disruption are potential contributors to a high prevalence of delirium in the ICU.^[^ ^22^, ^23^, ^24^, ^25^, ^26^, ^27^ ^]^ Also, delirious mice show reduced expression of *Per2* (a circadian clock component) in the SCN and hippocampus and application of the PER2 enhancer nobiletin attenuates the delirium‐like syndrome.^[^ ^27^ ^]^ However, the molecular mechanism by which circadian rhythms impact delirium is poorly understood.

Here, we show that mice with circadian disruption are delirious prone with aggravated cognitive impairment. We further disclose that deficiency of hippocampal *E4bp4*, as an output of circadian disruption, underlies delirium‐associated cognitive decline. Deficiency of *E4bp4* activates the ERK1/2 cascade via derepression of *Mapk1/3* transcription, leading to microglial activation and thus to compromised long‐term potentiation in the hippocampus and impaired cognition. Our findings define a causal role of clock dysfunction in delirium development and indicate *E4bp4* as a regulator of cognition at the crosstalk between circadian clock and delirium.

## 2 Results

### 2.1 Disruption of Circadian Rhythms in Delirious Mice and Patients With Delirium

**Figure 1:**
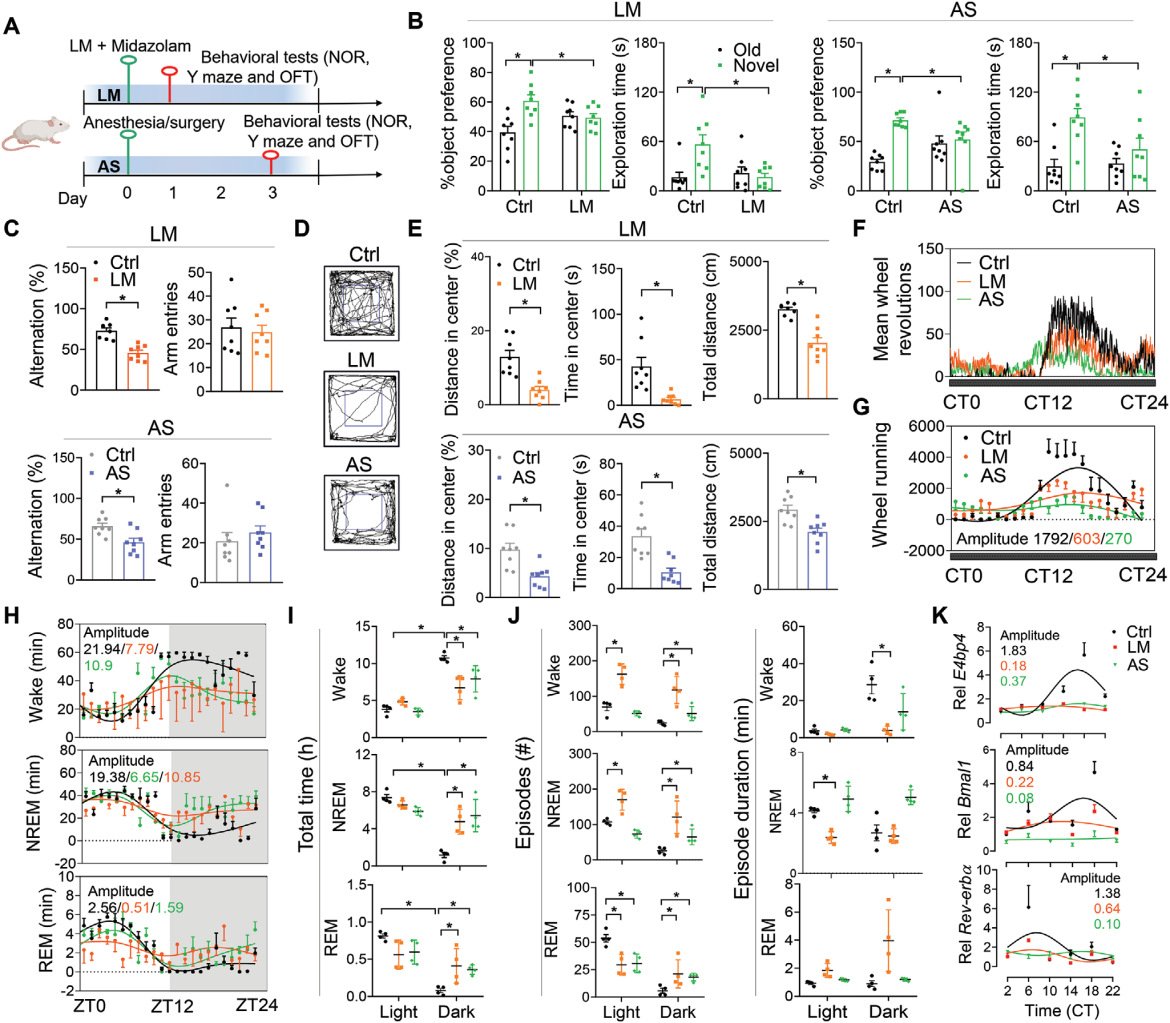
Disruption of circadian rhythms in mice with delirium‐like syndrome. A) Graphic illustration of the experimental procedures for behavioral tests. Note that LM and AS treatments were performed at day 0. Red circle indicates the time when behavioral tests were performed. B) Object preference for three groups of mice based on the NOR test, which was examined at day 1 (for LM) or day 3 (for AS) after delirium induction (*n* = 8 mice). C) Spontaneous alterations for three groups of mice based on the Y maze test, which were measured at day 1 (for LM) or day 3 (for AS) after delirium induction (*n* = 8 mice). D) Representative locomotor paths for three groups of mice in the OFT test. E) Locomotor activities for three groups of mice based on an OFT test, which were measured at day 1 (for LM) or day 3 (for AS) after delirium induction (*n* = 8 mice). F) Circadian wheel‐running activities recorded at a 1‐min interval for delirious and control mice (*n* = 4). G) Circadian wheel‐running activities recorded at a 60‐min interval for delirious and control mice (*n* = 4). H) Daily patterns of wake, NREM, and REM sleep time for delirious and control mice (*n* = 4). I) Total time of wake, NREM, and REM sleep in the light and dark phases (*n* = 4 mice). J) The number of episodes and episode duration for wake, NREM, and REM sleep in the light and dark phases (*n* = 4 mice). K) Relative expression of clock genes in the hippocampus from delirious and control mice (*n* = 4). Data are mean ± SEM. *p < 0.05 (*t* test or two‐way ANOVA with Bonferroni post hoc test). Rel: relative.

Mice were treated with lipopolysaccharide (LPS) plus midazolam (named LM treatment) or subjected to a simple laparotomy under isoflurane anesthesia (named AS treatment) to induce delirium‐like syndrome (**Figure** **1**A).^[^ ^27^, ^28^, ^29^ ^]^ Delirium‐associated cognitive deficits were assessed based on behavioral tests with novel object recognition (NOR) and Y maze. As expected, mice with LM or AS treatment showed reduced novel object preference in the NOR test and decreased spontaneous alternation in Y maze test, indicating impairment of cognitive function (Figure 1B,C; Figure S1, Supporting Information). Based on an open field test (OFT), they also had a reduced locomotor activity (decreased total distance), reflecting of delirium‐related behavioral disruption, such as lethargy and anxiety (Figure 1D,E). It was noted that the behavioral deficits recovered 6 days after LM treatment and 12 days after AS treatment (Figure S2, Supporting Information). We next tested whether circadian rhythms are affected in the delirium conditions. Mice with LM‐ and AS‐induced delirium showed attenuated circadian rhythms in wheel‐running activities with an increased activity in the subjective daytime and a reduced activity in the subjective nighttime (Figure 1F,G; Figure S3A, Supporting Information). The activity amplitudes of delirious mice were reduced to 15–34% of normal mice (Figure 1G). Moreover, circadian sleep–wake behavior was disrupted in delirious mice (Figure 1H,I; Figure S3B, Supporting Information). Delirious mice showed shortened wake time and prolonged sleep (i.e., NREM and REM) time in the dark phase (Figure 1I), and had altered sleep architecture with increased sleep fragmentation in the dark phase (Figure 1J). Supporting this, the circadian pattern of plasma corticosterone, a sleep‐related hormone, was disrupted in delirious mice (Figure S3C, Supporting Information). As the hippocampus has a major regulatory role in cognitive function, we examined circadian expression of clock genes in delirious hippocampus. Delirium resulted in overall disruption of core clock genes in mouse hippocampus, including *E4bp4, Bmal1, Rev‐erbα, Cry1*, and *Per2* (Figure 1K; Figure S3D, Supporting Information). Of note, *E4bp4* was one of the genes altered the most and its amplitude was reduced to 10–20% of the control (Figure 1K). In addition, we observed disrupted rhythms in *E4BP4, BMAL1*, and *REV‐ERBα* in the whole blood of patients with delirium (Figure S3E,F, Supporting Information). Altogether, delirium is associated with disrupted circadian rhythms and clock dysfunction in the hippocampus.

### 2.2 Circadian Disruption Sensitizes Mice to Delirium‐Like Syndrome

**Figure 2:**
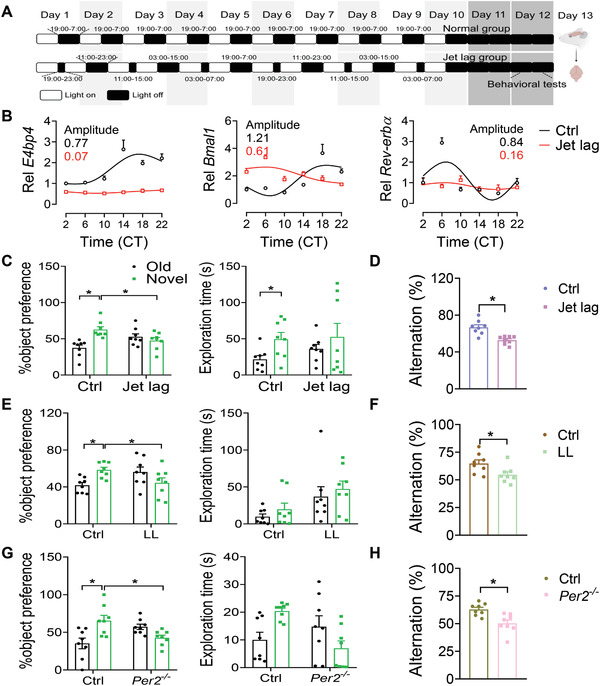
Circadian disruption sensitizes mice to delirium‐associated cognitive dysfunction. A) Experimental scheme for establishment of jet‐lagged mice. B) Relative expression of clock genes in the hippocampus from jet‐lagged and control mice (*n* = 4). C) Object preference for jet‐lagged and control mice based on the NOR test (*n* = 8 mice). D) Spontaneous alterations for jet‐lagged and control mice based on the Y maze test (*n* = 8 mice). E) Object preference for mice exposed 14 days to constant lighting based on the NOR test (*n* = 8 mice). F) Spontaneous alterations for mice exposed 14 days to constant lighting based on the Y maze test (*n* = 8 mice). G) Object preference for *Per2* ^−/−^ and control mice based on the NOR test (*n* = 8 mice). H) Spontaneous alterations for *Per2* ^−/−^ and control mice based on the Y maze test (*n* = 8 mice). *p < 0.05 (*t* test or two‐way ANOVA with Bonferroni post hoc test). LL: constant lighting.

Given that delirium is associated with disrupted circadian rhythms, it was of interest to investigate a potential role of circadian rhythms in the development of delirium. To this end, jet‐lagged mice (a model of physiological disruption of circadian rhythms) were established using a published protocol (i.e., 8 h light advance every 2 days for 10 days, **Figure** **2**A),^[^ ^30^ ^]^ and subjected to delirium assessment (on day 12). Jet lag resulted in perturbance of clock genes such as *E4bp4, Bmal1*, and *Rev‐erbα* in mouse hippocampus (Figure 2B). Intriguingly, jet‐lagged mice showed delirium‐like syndrome as evidenced by reduced novel object preference in the NOR test and decreased spontaneous alternation in Y maze test (Figure 2C,D; Figure S4, Supporting Information). We further tested the effects of constant lighting (for 14 days, another model of physiological circadian disruption) on delirium development. Constant lighting led to impaired hippocampus‐dependent cognitive performance as measured by the NOR and Y maze tests (on day 15) (Figure 2E,F; Figure S4, Supporting Information). Moreover, we examined the effects of *Per2* deletion (a genetic model of circadian disruption) on the development of delirium. *Per2‐*deficient (*Per2^−/−^*) mice showed an increased susceptibility to cognitive impairment according to the NOR and Y maze tests (Figure 2G,H; Figure S4, Supporting Information). Taken together, physiological and genetic circadian disruptions sensitize mice to delirium‐like syndrome with respect to cognitive dysfunction.

### 2.3 *E4bp4* Regulates Delirium‐Associated Cognitive Decline in a Circadian Time‐Dependent Fashion

**Figure 3:**
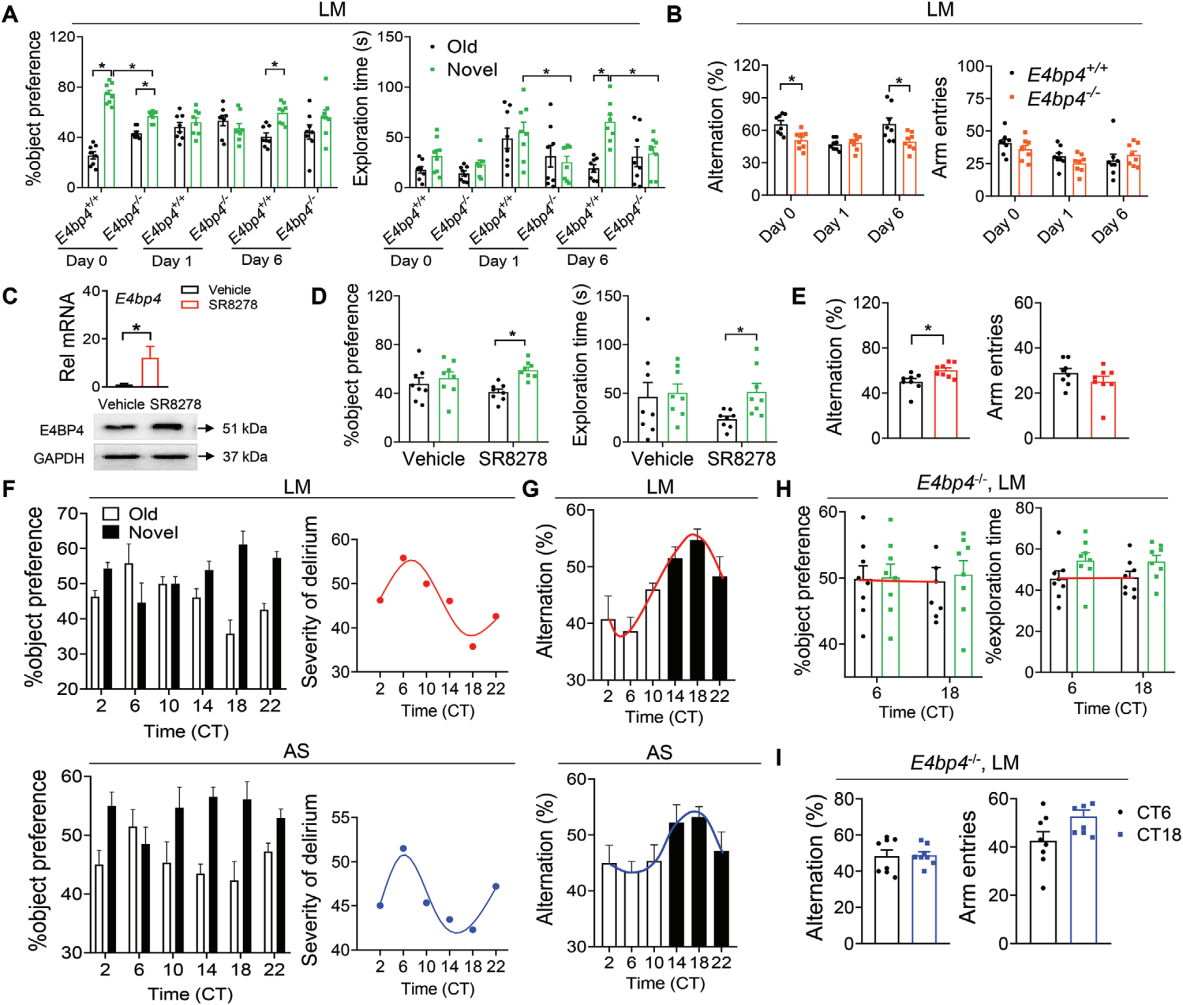
E4BP4 regulates delirium in a circadian time‐dependent fashion. A) Object preference for *E4bp4* ^−/−^ and control mice based on the NOR test, which was examined before (day 0) and at days 1 and 6 after delirium induction by LM. B) Spontaneous alterations for *E4bp4* ^−/−^ and control mice based on the Y maze test, which were examined before (day 0) and at days 1 and 6 after delirium induction by LM. C) Effects of SR8278 (injected into the hippocampus) on hippocampal E4BP4 mRNA and protein in mice (*n* = 3). D) Object preference for SR8278‐treated and control mice based on the NOR test, which was examined at day 1 after delirium induction by LM. E) Spontaneous alterations for SR8278‐treated and control mice based on the Y maze test, which were examined at day 1 after delirium induction by LM. F) Circadian changes in object preference for LM and AS‐treated mice. The severity of delirium was derived as the percent of old object preference. G) Circadian changes in spontaneous alterations for LM and AS‐treated mice. H) Object preference for *E4bp4* ^−/−^ mice examined at CT6 and CT18 after LM treatment according to the NOR test. I) Spontaneous alterations for *E4bp4* ^−/−^ mice examined at CT6 and CT18 after LM treatment according to the Y maze test. In all panels except C, data are mean ± SEM (*n* = 8). *p <0.05 (*t* test or two‐way ANOVA with Bonferroni post hoc test).

As shown above (Figure 1K; Figure S3E,F, Supporting Information), *E4bp4* expression was severely compromised in the hippocampus of delirious mice and in the whole blood of humans with delirium. On the other hand, we observed marked disruption of *E4bp4* in the hippocampus of jet‐lagged mice (Figure 2B). It was thus hypothesized that *E4bp4* has a critical role in connecting delirium to circadian clock. To test this hypothesis, we examined the regulatory effects of *E4bp4* on delirium using mice with global deletion of *E4bp4* (*E4bp4^−/−^* mice). *E4bp4^−/−^* mice showed exacerbated delirium‐associated cognitive decline after LM or AS induction, as evidenced by a slower recovery from behavioral deficits based on NOR and Y maze tests (**Figure** **3**A,B; Figure S5, Supporting Information). Furthermore, application of SR8278, a chemical that can increase E4BP4 expression in the hippocampus by antagonizing its repressor REV‐ERB*α*,^[^ ^31^ ^]^ rescued LM‐induced delirium‐associated cognitive deficits (Figure 3C–E). These findings indicated a regulatory role of *E4bp4* in the development of delirium in mice. As *E4bp4* is rhythmically expressed in mouse hippocampus with higher expression in the subjective nighttime (Figures 1K and 2B), it was of interest to test circadian time‐varying severity in delirium symptoms. The severity of delirium (or the extent of cognitive impairment, negatively correlated with the novel object preference in the NOR test and with spontaneous alternation in Y maze test) was assessed after LM or AS induction at six different circadian time points (i.e, CT2, CT6, CT10, CT14, CT18, and CT22). Delirium severity displayed a robust circadian rhythm (most severe at CT6 and least severe at CT18) (Figure 3F,G). However, the circadian time‐dependency of delirium severity was lost in *E4bp4^−/−^* mice (Figure 3H,I). Altogether, *E4bp4* regulates the development of delirium with respect to cognitive impairment, accounting for circadian rhythm in the disease severity.

### 2.4 Microglial Activation in the Hippocampus Underlies Delirium‐Associated Cognitive Impairment in Mice

**Figure 4:**
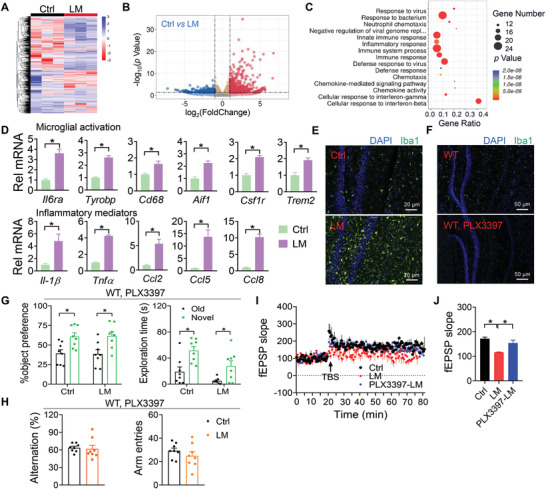
Hippocampal microglial activation underlies delirium‐associated cognitive impairment in mice. A) Heatmap for differentially expressed genes (DEGs) caused by delirium. B) Volcano plot showing differential gene expression. C) GO enrichment analysis of DEGs associated with delirium. D) Relative expression of transcripts related to microglial activation in the hippocampus of delirious (induced by LM) and control mice at day 1 (*n* = 4). E) Immunofluorescent staining of hippocampus from delirious and control mice using the microglial marker Iba1 (green) and DAPI (blue). Scale bar, 20 µm. F) Immunofluorescent staining of hippocampus from PLX3397‐treated and control mice using the microglial marker Iba1 (green) and DAPI (blue). Scale bar, 50 µm. G) Object preference for PLX3397‐treated mice at day 1 after delirium induction by LM based on the NOR test (*n* = 8). H) Spontaneous alterations for PLX3397‐treated mice at day 1 after delirium induction by LM based on the Y maze test (*n* = 8). I) Field excitatory postsynaptic potential (fEPSP) slope (normalized to baseline) recorded over time for hippocampal slices from delirious and control mice (*n* = 3). J) Normalized fEPSP slope (last 20 min) for hippocampal slices from delirious and control mice (*n* = 3). Data are mean ± SEM. *p < 0.05 (*t* test). TBS, theta burst stimulation. PLX3397‐LM, PLX3397‐treated mice after LM induction.

To understand the pathological basis underlying the delirium‐associated cognitive impairment, we performed transcriptomic analyses of the hippocampus from delirious mice (induced by LM) and controls collected at CT6 when the disease is the most severe (Figure 3). A total of 1032 differentially expressed genes (DEGs) were identified between delirious and control hippocampus (**Figure** **4**A). Of these DEGs, 728 genes were up‐regulated and 302 genes down‐regulated (Figure 4B). Gene ontology (GO) enrichment analyses suggested an association of delirium with activation of immune and inflammatory responses (Figure 4C). As microglia and astrocytes are key regulators of neuroinflammation,^[^ ^32^, ^33^ ^]^ we wondered whether they are affected in delirious mice. LM‐ and AS‐induced delirium led to microglial activation in the hippocampus as evidenced by up‐regulation of microglial activation‐related genes (such as *Il6ra*, *Tyrobp*, *Cd68*, *Aif1*, *Csf1r*, and *Trem2)* and of relevant inflammatory factors (such as *Il‐1β, Tnfα*, *Ccl2, Ccl5*, and *Ccl8*) (Figure 4D; Figure S6A, Supporting Information). This was supported by an increased number of Iba1^+^ cells according to immunofluorescent staining (Figure 4E; Figure S6B,C, Supporting Information). By contrast, astrocytes were unaffected in the hippocampus of delirious mice as illustrated by no significant changes in astrocyte activation markers (such as *Gfap* and *Timp1*) or in the number of GFAP^+^ cells (Figure S6D–F, Supporting Information). Given that the delirious condition was associated with microglial activation, we further tested whether microglia have a role in delirium‐associated cognitive impairment. To this end, microglia were depleted by feeding mice (wild‐type) with PLX3397 for 14 days and cognitive function was assessed after delirium induction. Unsurprisingly, mice treated with PLX3397 showed a marked loss of microglia (Figure 4F; Figure S6G, Supporting Information). We found no significant inflammation in the hippocampus of PLX3397‐treated mice after LM induction (i.e., no changes in inflammatory factors such as *Tnfα*, *Il‐1β*, *Il‐6* and *Ccl2;* Figure S6H,I, Supporting Information). Intriguingly, microglia depletion protected mice from cognitive decline associated with LM‐induced delirium, as evidenced by their normal cognitive behaviors (i.e., novel object preference and spontaneous alternation) in the NOR and Y maze tests (Figure 4G,H). Activation of microglia has been demonstrated in the literature to impair the hippocampal long‐term potentiation (LTP), a neural basis of cognitive function.^[^ ^34^ ^]^ We found that LTP was attenuated in the hippocampus of delirious mice (Figure 4I,J). However, microglia depletion by PLX3397 prevented delirium‐induced LTP impairment in mice (Figure 4I,J). Altogether, these findings indicated that microglial activation in the hippocampus contributes to delirium‐associated cognitive impairment in mice through impairing hippocampal LTP.

### 2.5 *E4bp4* Regulates Delirium‐Associated Cognitive Decline Through Limiting Microglial Activation in Mouse Hippocampus

**Figure 5:**
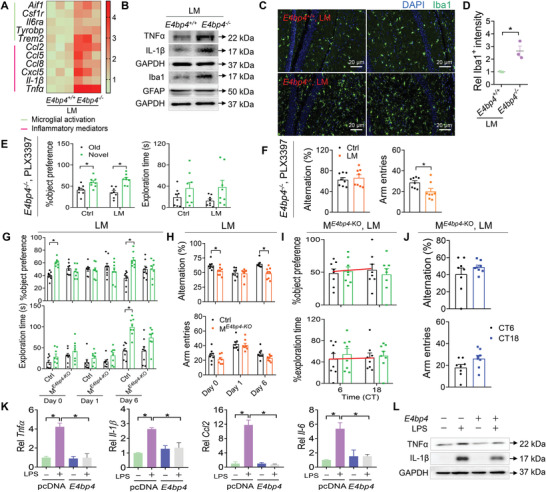
*E4bp4* deficiency promotes microglial activation and inflammation in the hippocampus of delirious mice. A) Relative expression of transcripts related to microglial activation in the hippocampus of *E4bp4* ^−/−^ and control mice at day 1 after LM treatment. B) Relative levels of proteins related to microglial activation in the hippocampus of *E4bp4* ^−/−^ and control mice at day 1 after LM treatment (*n* = 4). C) Immunofluorescent staining of hippocampus from *E4bp4* ^−/−^ and control mice at day 1 after LM treatment. The left images present the dentate gyrus of hippocampus and its periphery, whereas the right present the CA1 area of hippocampus and its periphery. Scale bar, 20 µm. D) Quantification of Iba1^+^ cells in the hippocampus of mice (*n* = 3). E) Object preference for PLX3397‐treated *E4bp4* ^−/−^ mice at day 1 after delirium induction by LM based on the NOR test (*n* = 8). F) Spontaneous alterations for PLX3397‐treated *E4bp4* ^−/−^ mice at day 1 after delirium induction by LM based on the Y maze test (*n* = 8). G) Object preference for M*^E4bp4–KO^* (microglia*^E4bp4–KO^*) and control mice based on the NOR test, which was examined before (day 0) and at days 1 and 6 after delirium induction by LM. H) Spontaneous alterations for M*^E4bp4–KO^* and control mice based on the Y maze test, which were examined before (day 0) and at days 1 and 6 after delirium induction by LM. I) Object preference for M*^E4bp4–KO^* mice examined at CT6 and CT18 after LM treatment according to the NOR test. J) Spontaneous alterations for M*^E4bp4–KO^* mice examined at CT6 and CT18 after LM treatment according to the Y maze test. K) mRNA levels of inflammatory factors in *E4bp4*‐overexpressed BV2 cells after LPS stimulation for 8 h (*n* = 4). L) Protein levels of IL‐1β and TNFα in *E4bp4*‐overexpressed BV2 cells after LPS stimulation for 8 h (*n* = 4). In all panels except A, B, C and L, data are mean ± SEM. *p < 0.05 (*t* test or two‐way ANOVA with Bonferroni post hoc test).

As microglial activation is involved in pathogenesis of delirium and *E4bp4* has a potential role in immune responses,^[^ ^19^, ^20^ ^]^ we next examined whether *E4bp4* regulates delirium via modulation of microglial activation. As noted before, *E4bp4* ^−/−^ mice fed a normal chow diet showed exacerbated delirium‐associated cognitive impairment (Figure 3A,B). This was accompanied by microglial activation in the hippocampus as the levels of functional markers (e.g., *Aif1*, *Csf1r*, and *Trem2*) were elevated and the number of Iba1^+^ cells were increased after delirium induction (**Figure** **5**A–D; Figure S7, Supporting Information). However, *E4bp4* ^−/−^ mice fed a chow diet containing PLX3397 (used to deplete microglia) were deficient in microglial activation and lack of inflammatory responses in the hippocampus (Figure S8, Supporting Information), and were protected from delirium‐associated cognitive impairment (Figure 5E,F). These findings suggested involvement of microglial activation in *E4bp4* regulation of cognitive dysfunction in delirium‐like syndrome. Supporting this, mice with *E4bp4* specifically deleted in microglia (generated by breeding mice carrying a conditional *E4bp4* allele with mice expressing *Cx3cr1^CreER^*, named microglia*^E4bp4‐KO^* mice^[^ ^35^ ^]^) showed aggravated delirium‐associated cognitive decline, as evidenced by a slower recovery based on the NOR and Y maze tests (Figure 5G,H). In addition, the circadian time‐dependency of delirium severity was lost in microglia*^E4bp4‐KO^* mice (Figure 5I,J). In contrast, mice lacking in *E4bp4* in hippocampal CA1 neurons (*E4bp4^cKO^* mice) had a normal phenotype (Figure S9, Supporting Information). Moreover, *E4bp4* showed inhibitory effects on LPS‐induced inflammation in a microglial cell line (BV2 cells) as it decreased *Tnfα*, *Il‐1β*, *Il‐6* and *Ccl2* mRNAs as well as TNF*α* and IL‐1β proteins (Figure 5K,L). Prior studies suggest that neuronal loss, astroglial activation, down‐regulation of brain‐derived neurotrophic factor (BDNF), disruption of blood–brain barrier, and brain hypoxia contribute to delirium pathophysiology.^[^ ^36^, ^37^, ^38^, ^39^, ^40^ ^]^ We tested whether these factors are involved in *E4bp4* regulation of delirium. The numbers of neurons and astrocytes did not differ between *E4bp4* ^−/−^ and control mice (Figure S10A,B, Supporting Information). We found no changes either in BDNF and its receptor TrkB, tight junction‐associated proteins (ZO‐1 and occludin), as well as *Hif1α* (hypoxia‐induced factor) and its target genes such as *Phd2* (*oxygen‐sensing prolyl hydroxylase domain protein 2*) and *Vegfa* (*vascular endothelial growth factor A*) (Figure S10C–E). Taken together, microglial activation is likely the main mechanism for regulation of delirium‐associated cognitive decline by *E4bp4*.

### 2.6 *E4bp4* Restrains Microglial Activation Via ERK1/2 Signaling in the Hippocampus

**Figure 6:**
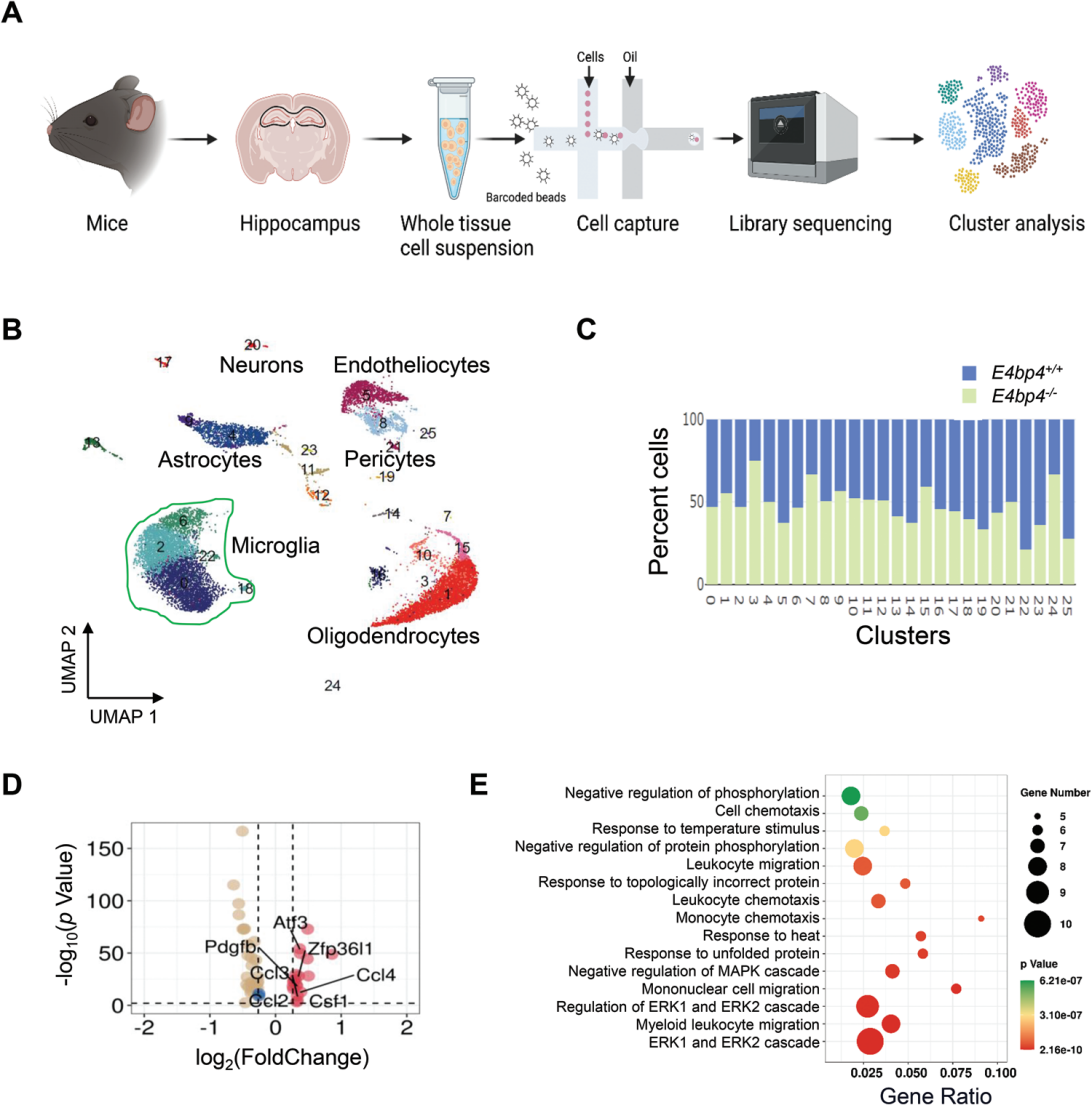
Single‐cell RNA sequencing reveals a potential role of ERK1/2 signaling in E4BP4 regulation of hippocampal microglial activation. A) Schematic workflow for 10× genomics single‐cell RNA sequencing of the hippocampus from *E4bp4* ^−/−^ and control mice. B) Visualization of six major classes of cells using uniform manifold approximation and projection (UMAP). The numbers represent cell clusters, colored according to density clustering. Each dot is a single cell, and cells are laid out to show similarities. C) Cell distribution for each cluster. Green bar denotes the percent of cells for *E4bp4* ^−/−^ libraries and blue bar denotes the percent of cells for control libraries. D) Differential gene expression between *E4bp4* ^−/−^ and control mice. E) *E4bp4*‐associated differentially expressed genes, converging on ERK1/2 signaling and related pathways based on GO enrichment analysis.

To explore the mechanisms by which *E4bp4* regulates microglial activation and inflammation in the hippocampus, we performed single‐cell RNA‐sequencing of hippocampus tissues from *E4bp4* ^−/−^ and control mice (**Figure** **6**A). A total of 22 259 cells were sequenced based on 10× Genomics libraries of *E4bp4* ^−/−^ and control hippocampus, of which 14 803 cells passed the quality filters and the average number of detected genes per cells was about 1800 (Figure S11A–D, Supporting Information). 25 clusters were identified and grouped into six distinct cell types (i.e., microglia, astrocytes, oligodendrocytes, endotheliocytes, neurons, and pericytes) based on known cell identity markers (Figure 6B; Figure S11E, Supporting Information).^[^ ^41^ ^]^ Each of the cell clusters from *E4bp4* ^−/−^ and control libraries had a similar proportion of cells, suggesting that *E4bp4* ablation did not affect the cell types (Figure 6C; Figure S11F, Supporting Information). Notably, microglia were one of the most abundant cell populations, present in clusters 0, 2, 6, 18, and 22 (Figure 6B). Thus, we analyzed the DEGs in hippocampal microglia between *E4bp4* ^−/−^ and control mice. Of 52 DEGs, the inflammatory factors such as *Ccl2* and *Ccl4* as well as the microglial activation transcript *Csf1* were significantly increased, suggesting microglial activation in the gene knockout mice (Figure 6D). Supporting this, qPCR analyses and immunofluorescent staining demonstrated increases in microglial activation transcripts and in Iba1^+^ cell number in the hippocampus of *E4bp4* ^−/−^ mice (Figure S12, Supporting Information). According to GO enrichment analyses, *E4bp4*‐associated DEGs converged on ERK1/2 signaling and related pathways (such as ERK1/2 cascade, regulation of ERK1/2 cascade, negative regulation of MAPK cascade, and negative regulation of protein phosphorylation), suggesting a potential role of ERK1/2 signaling in *E4bp4* regulation of hippocampal microglial activation (Figure 6E). In fact, it has been established that ERK1/2 signaling pathway contributes to microglial activation and ensuing inflammatory responses.^[^ ^42^, ^43^ ^]^ The underlying mechanism involves activation of NF‐*κ*B signaling, an essential regulator of microglial activation and neuroinflammation.^[^ ^44^, ^45^ ^]^

**Figure 7:**
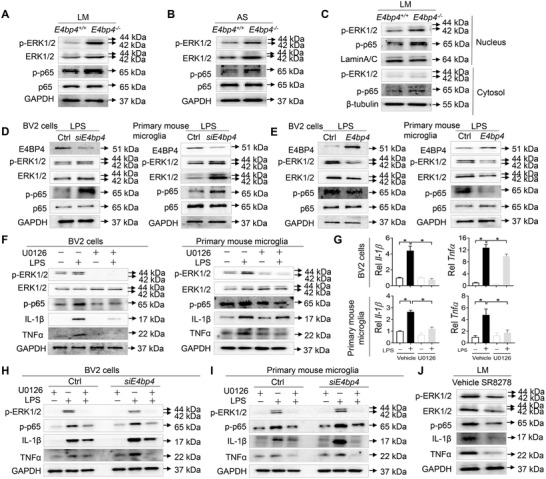
*E4bp4* regulates microglial activation via ERK1/2 signaling in the hippocampus. A) Effects of *E4bp4* loss on total p‐ERK1/2, ERK1/2, p65, and p‐p65 proteins in the hippocampus of mice at day 1 after LM treatment. B) Effects of *E4bp4* loss on total p‐ERK1/2, ERK1/2, p65, and p‐p65 proteins in the hippocampus of mice at day 3 after AS treatment. C) Effects of *E4bp4* loss on nuclear p‐ERK1/2, ERK1/2, p65, and p‐p65 proteins in the hippocampus of mice at day 1 after LM treatment. D) Effects of *E4bp4* knockdown on p‐ERK1/2, ERK1/2, p65, and p‐p65 proteins in BV2 cells and in primary mouse microglia. Cells were transfected with si*E4bp4* or control for 40 h followed by treatment with LPS or vehicle for 8 h. E) Effects of *E4bp4* overexpression on p‐ERK1/2, ERK1/2, p65, and p‐p65 proteins in BV2 cells and in primary mouse microglia. Cells were transfected with *E4bp4* plasmid or control for 40 h followed by treatment with LPS or vehicle for 8 h. F) Effects of U0126 on p‐p65, IL‐1*β*, and TNF*α* proteins in BV2 cells and in primary mouse microglia. Cells were pretreated with U0126 for 1 h followed by treatment with LPS or vehicle for 8 h. G) Effects of U0126 on *Il‐1β* and *Tnfα* mRNAs in BV2 cells and in primary mouse microglia. Cells were pretreated with U0126 for 1 h followed by treatment with LPS or vehicle for 8 h. H) Effects of U0126 on p‐p65, IL‐1*β*, and TNF*α* proteins in *E4bp4‐*deficient BV2 cells. Transfected cells were pretreated with U0126 for 1 h followed by treatment with LPS or vehicle for 8 h. I) Effects of U0126 on p‐p65, IL‐1*β*, and TNF*α* proteins in *E4bp4‐*deficient primary mouse microglia. Transfected cells were pretreated with U0126 for 1 h followed by treatment with LPS or vehicle for 8 h. J) Effects of SR8278 on p‐ERK1/2, ERK1/2, p‐p65, IL‐1*β*, and TNF*α* proteins in the hippocampus of mice at day 1 after LM treatment. In panel G, data are mean ± SEM. *p < 0.05 (*t* test or two‐way ANOVA with Bonferroni post hoc test).

We next examined whether *E4bp4* does regulate ERK1/2 signaling pathway in the hippocampus. *E4bp4* ^−/−^ mice after delirium induction showed increases in total phosphorylated proteins of ERK1/2 and p65 (an NF‐*κ*B subunit and a downstream target of ERK1/2) in the hippocampus (**Figure** **7**A,B), and an elevation in nuclear phosphorylated ERK1/2 and p65 (Figure 7C). Furthermore, *E4bp4* ablation led to an increase in phosphorylation of both ERK1/2 and p65 in BV2 cells and primary mouse hippocampal microglia (Figure 7D). Consistently, *E4bp4* overexpression resulted in reduced phosphorylation of ERK1/2 and p65 in the cells (Figure 7E). As expected, U0126, an ERK phosphorylation inhibitor, markedly alleviated LPS (an NF‐*κ*B activator)‐induced inflammation (measured by TNFα and IL‐1β levels) in BV2 cells and primary mouse hippocampal microglia (Figure 7F,G). Intriguingly, in the presence of U0126, the effects of *E4bp4* ablation on LPS‐induced inflammation were attenuated in BV2 cells and primary mouse hippocampal microglia (Figure 7H,I). Additionally, SR8278 (a chemical that can increase E4BP4 expression, Figure 3C) decreased the levels of phosphorylated ERK1/2 and p65 in mouse hippocampus, accompanied by alleviated inflammation (Figure 7J). Altogether, these findings indicated that *E4bp4* regulates ERK1/2 signaling pathway and inflammation in the hippocampus.

### 2.7 E4BP4 Represses the Transcription of *Mapk1* and *Mapk3*, Two Genes Respectively Encoding ERK2 and ERK1 Proteins

**Figure 8:**
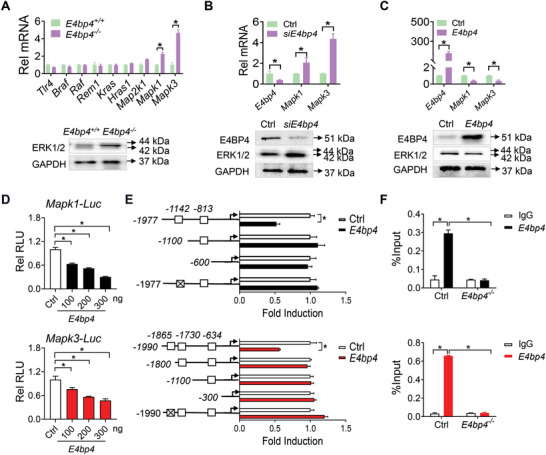
E4BP4 regulates the transcription of *Mapk1* and *Mapk3*, two genes respectively encoding ERK2 and ERK1 proteins. A) Effects of *E4bp4* loss on the expression of genes involved in ERK1/2 cascade in the hippocampus of mice (*n* = 4). B) Effects of *E4bp4* knockdown on ERK1/2 expression in BV2 cells (*n* = 4). C) Effects of *E4bp4* overexpression on ERK1/2 expression in BV2 cells (*n* = 4). D) *E4bp4* dose‐dependently inhibits *Mapk1* and *Mapk3* promoter activities (*n* = 6). E) Effects of *E4bp4* on the activities of *Mapk1* and *Mapk3* luciferase reporters (truncated and mutated versions) (*n* = 6). F) ChIP assays showing recruitment of E4BP4 protein to the D‐boxes of *Mapk1* and *Mapk3* in the hippocampus of control mice. Note that such recruitment does not exist in the hippocampus of *E4bp4* ^−/−^ mice (*n* = 4). Data are mean ± SEM. *p < 0.05 (one‐way or two‐way ANOVA with Bonferroni post hoc test).

Next, we investigated the mechanisms by which *E4bp4* regulates ERK1/2 signaling pathway in the hippocampus. We first tested whether E4BP4 affects the phosphorylation of MEK1/2, the MAPKK kinases that phosphorylate and activate ERK1/2.^[^ ^46^ ^]^ We observed unchanged phosphorylation of MEK1/2 in the hippocampus of *E4bp4* ^−/−^ mice (Figure S13A, Supporting Information). Autophagy proteins as cellular scaffolds play a regulatory role in ERK phosphorylation.^[^ ^47^ ^]^ We found no changes in hippocampal expression of autophagy proteins such as ATG7 and LC3 in *E4bp4* ^−/−^ mice (Figure S13B, Supporting Information). Thus, the roles of MEK1/2 and autophagy proteins in *E4bp4* regulation of ERK1/2 can be excluded. As *E4bp4* is known as a transcriptional repressor, we further examined whether E4BP4 regulates the transcription and expression of *Mapk1* and *Mapk3* (two genes encoding ERK2 and ERK1 protein, respectively). Loss of *E4bp4* in mice (without delirium induction) significantly increased *Mapk1*/*3* mRNAs and ERK1/2 proteins in the hippocampus (**Figure** **8**A). Similar alterations in both *Mapk1*/*3* mRNAs and ERK1/2 proteins were observed in *E4bp4* ^−/−^ mice with delirium (Figure 7A,B; Figure S13C, Supporting Information). Notably, other genes involved in ERK1/2 cascade, including *Tlr4, Braf, Raf, Rem1, Kras, Hras1* and *Map2k1*, were unaffected in *E4bp4* knockout mice (Figure 8A; Figure S13C, Supporting Information). Moreover, knockdown of *E4bp4* (by a specific siRNA) led to increases in *Mapk1*/*3* mRNAs and ERK1/2 levels in BV2 cells (Figure 8B), whereas overexpression of *E4bp4* resulted in reduced expression of *Mapk1*/*3* and their proteins (Figure 8C). These data support a negative role of E4BP4 in regulation of ERK1/2 expression in hippocampal microglia.

Parallel changes in *Mapk1/3* mRNAs and in their proteins suggested a transcriptional mechanism for E4BP4 regulation of ERK1/2. In luciferase reporter assays, E4BP4 dose‐dependently inhibited the promoter activities of both *Mapk1* and *Mapk3* (Figure 8D). Sequence analysis revealed two potential D‐box (a putative DNA motif for E4BP4 binding and function) elements in *Mapk1* promoter and three potential D‐boxes in *Mapk3* promoter (Figure 8E). Furthermore, truncation and mutation assays demonstrated that −1142 bp D‐box in the *Mapk1* and −1865 bp D‐box in the *Mapk3* promoter were responsible for E4BP4 effects (Figure 8E). According to chromatin immunoprecipitation (ChIP) assays, E4BP4 protein was recruited to the −1142 bp D‐box of *Mapk1* and to the −1865 bp D‐box of *Mapk3* in mouse hippocampus (Figure 8F). However, recruitment of E4BP4 to *Mapk1*/*3* ceased to exist in *E4bp4‐*deficient hippocampus (Figure 8F). Taken together, E4BP4 trans‐repressed *Mapk1* and *Mapk3* via direct binding to a D‐box element in their promoters.

## 3 Discussion

We have observed perturbance of circadian rhythms in the behaviors (such as the locomotor activity and sleep–wake cycle) and clock gene expression in mice with delirium‐like syndrome, consistent with our and previous clinical observations that patients with delirium show disrupted circadian rhythms (Figure S3E/F).^[^ ^22^, ^23^, ^24^, ^25^, ^26^ ^]^ Importantly, we show that mice with circadian disruption are delirious prone with aggravated cognitive impairment, supporting involvement of clock dysfunction in the pathogenesis of delirium. Further, we disclose that deficiency of hippocampal *E4bp4*, as an output of circadian disruption, underlies delirium‐associated cognitive impairment. Deficiency of *E4bp4* activates the ERK1/2 cascade via derepression of *Mapk1/3* transcription, leading to microglial activation and thus to compromised hippocampal LTP (synaptic plasticity), a key neural basis for cognition. The working mechanism for microglia regulation of LTP activity and cognitive function has been established in the literature.^[^ ^34^ ^]^ Activation of microglia produces IL‐1*β* and TNF*α*, which impairs induction of LTP and memory performance by preventing AMPA receptor GluR1 subunit phosphorylation.^[^ ^48^ ^]^ Therefore, we propose that hippocampal *E4bp4* coordinates circadian clock regulation of delirium‐associated cognitive decline, explaining why circadian disruption (e.g., a problem commonly shared by ICU and elderly patients) promotes the occurrence of delirium.

Although we have uncovered the molecular mechanism underlying circadian regulation of cognitive dysfunction in delirium‐like syndrome, why circadian rhythms are disrupted in the delirium models is unaddressed. As delirium is associated with neuroinflammation in the brain,^[^ ^49^ ^]^ it is speculated that delirium‐induced disruption of circadian rhythms is, at least in part, attributed to neuroinflammation as the inflammation regulators (e.g., NF‐*κ*B) and inflammatory cytokines (e.g., TNF*α* and IL‐1*β*) can regulate expression of core clock genes such as *Rev‐erbα*, *Pers*, and *Dbp*.^[^ ^50^, ^51^, ^52^ ^]^ We found that *E4bp4* is markedly down‐regulated in the hippocampus in delirium models. *E4bp4* is an output gene of circadian clockwork and is also regarded as a clock component.^[^ ^17^ ^]^ In fact, loss of *E4bp4* in mice led to disturbances in circadian behaviors such as the locomotor activity and sleep–wake cycle (Figure S14, Supporting Information). Hence, mutual interactions may exist between delirium and circadian clock (i.e., a crosstalk or bidirectional regulation phenomenon). This type of crosstalk would probably promote a vicious cycle between circadian disruption and delirium development, highlighting a critical role of circadian rhythms in delirium pathophysiology.

We found that the delirium is more severe in the daytime (rest phase for mice) than in the nighttime with respect to cognitive decline (Figure 3). This is in line with the clinical observation that delirium symptoms tend to be worse at night (rest phase for humans).^[^ ^53^ ^]^ The time‐varying severity of delirium in mice is well explained by the circadian expression of hippocampal *E4bp4* (as a delirium‐inhibiting gene) with lower expression during the rest period and higher expression during the activity period (Figures 1K and 3). The circadian patterns of *Mapk1/3* are antiphase to that of *E4bp4* in the hippocampus (Figure S15, Supporting Information), supporting E4BP4 as a transcriptional repressor of *Mapk1/3* and as a direct driver of *Mapk1/3* rhythms. Therefore, there may be a need to apply time‐based therapy and medical care for delirium due to a circadian feature of the disease.

We have established a causal relationship of the clock component *E4bp4* with microglial activation and delirium‐like syndrome, supporting an essential role of circadian clock in the regulation of microglial activity (known to follow a circadian rhythm^[^ ^54^ ^]^) and related psychiatric diseases. Combined with prior reports, circadian clock appears to regulate microglial activity using multiple components with distinct mechanisms, including activation of ERK1/2 cascade by *E4bp4*, enhancement of NF‐*κ*B signaling and synaptic phagocytosis by *Rev‐erbα*, and augmentation of phagocytic capacity by *Bmal1*.^[^ ^32^, ^35^, ^55^ ^]^ The finding that *E4bp4* restrains microglial activation and promotes hippocampus‐dependent cognitive performance helps to explain why mice with *Bmal1* specifically deleted in microglia are associated with improved long‐term memory and cognitive flexibility, and why the small molecule nobiletin can attenuate delirium‐like syndrome.^[^ ^27^, ^35^ ^]^ These findings are probably due to up‐regulation of *E4bp4* in the genetically modified mice as *Bmal1* is an indirect negative regulator of *E4bp4* and in nobiletin‐treated animals as nobiletin targets RORs (retinoid‐related orphan receptors) which are transcriptional activators of *E4bp4*.^[^ ^56^, ^57^ ^]^

Mouse models of delirium were established by treatment with LM and AS that resemble a variety of clinical insults (such as surgery, sepsis, trauma, infection, and deliriogenic medications) as previously described,^[^ ^3^, ^6^ ^]^ and demonstrate significant deficits in the locomotor activity, cognitive performance, and anxiety behavior, reminiscent of human delirium. Thus, these two animal models can be useful for elucidating cellular and molecular changes that may have a causal role in inducing the observed brain dysfunction, although it is difficult to incontrovertibly demonstrate the presence of delirium in rodents.^[^ ^6^ ^]^ It was noted that the behavioral deficits in delirium models recovered ~6–12 days after disease induction (Figure S2, Supporting Information). This is not surprising because delirium is usually reversible, and its duration is typically short lasting from a few days to weeks.^[^ ^6^ ^]^ Impaired attention is another core feature of delirium according to DSM‐5 diagnostic criteria.^[^ ^58^ ^]^ However, we did not explicitly assess the changes in the attention function in our delirium models, although inattention has been described in similar models.^[^ ^59^ ^]^ It remains unaddressed whether and how the circadian clock regulates delirium‐associated attention impairment.

Single‐cell RNA‐sequencing has been used to examine which types of cells in mouse hippocampus are affected by loss of *E4bp4* and to screen the altered cellular and molecular pathways. We found that the abundant cell populations in the hippocampus include microglia, oligodendrocytes, and astrocytes (Figure 6), consistent with a prior report.^[^ ^60^ ^]^ Notably, microglia were affected the most by *E4bp4* ablation due to a high number of DEGs, which converge on ERK1/2 signaling and related pathways (Figure 6). By contrast, limited numbers of DEGs were found for other cell types and no GO pathways can be enriched. Thus, these implicate that *E4bp4* regulates hippocampus function via modulating ERK1/2 signaling and microglial activity, which was validated by a series of subsequent in vitro and in vivo studies (Figures 7 and 8). It was proposed that *E4bp4* regulates *Mapk1/3* transcription to modulate ERK1/2 signaling and microglial activation, which is supported by the fact that *Mapk1/3* transcription and ERK1/2 expression are positively correlated with activation of ERK1/2 signaling.^[^ ^61^ ^]^

In summary, circadian disruption sensitizes mice to delirium with aggravated cognitive impairment due to down‐regulation of hippocampal *E4bp4*. Mechanistically, deficiency of *E4bp4* activates the ERK1/2 cascade via derepression of *Mapk1/3* transcription, leading to microglial activation and thus to compromised LTP and cognition. These findings indicate *E4bp4* as a regulator of cognition at the crosstalk between circadian clock and delirium, and as a therapeutic target for delirium.

## 4 Experimental Section

### Materials

LPS and granulocyte‐macrophage colony‐stimulating factor (GM‐CSF) were purchased from Sigma–Aldrich (St. Louis, MO). U0126 was purchased from MCE (Monmouth Junction, NJ). Midazolam was obtained from the First Affiliated Hospital of Jinan University (Guangzhou, China). PLX3397 chow (290 mg kg^−1^, formulated in AIN‐76A) was obtained from Plexxikon Inc. (Berkeley, CA). *Mapk1* luciferase reporters (−1977/+123 bp and two D‐box‐mutated versions) and *Mapk3* luciferase reporters (−1990/+100 bp and three D‐box‐mutated versions) were obtained from Tsingke Biotech (Beijing, China). pRL‐TK, pcDNA3.1, pcDNA3.1‐*E4bp4*, si*E4bp4* (siRNA targeting *E4bp4*), and a negative control for siRNAs (sequences provided in Table S1, Supporting Information) were obtained from Transheep Technologies (Shanghai, China).

### Animals

*E4bp4* ^−/−^ and *Per2* ^−/−^ (C57BL/6 background) mice had been established in the authors’ laboratory.^[^ ^62^, ^63^ ^]^ Microglia*^E4bp4‐KO^* mice were generated by breeding mice carrying a conditional *E4bp4* allele (i.e., exon 2 floxed allele, *E4bp4^fl/fl^*) mice with mice expressing *Cx3cr1^CreER^*. Genotyping of mice harboring the conditional *E4bp4* allele was performed with primers F: 5’‐TCAAAGTGGAGGCTTTGGAC‐3’ and R: 5’‐CACAAGGACACCCAGACAGA‐3’. Genotyping of mice expressing *Cx3cr1^CreER^* was performed with primers *Cx3cr1^CreER^*‐F: 5’‐AAG ACTCACGTGGACCTGCT‐3’, *Cx3cr1^CreER^*‐R1: 5’‐CGG TTATTCAACTTGCAC CA‐3’ and *Cx3cr1^CreER^*‐R2: 5’‐GG ATGTTGACTTCCGAG TTG‐3’. Mice were treated with tamoxifen (i.p., 20 mg mL^−1^) once daily for 3 days to delete the *E4bp4* allele via cre‐mediated recombination.^[^ ^35^ ^]^ Mice with *E4bp4* specifically deleted in the CA1 pyramidal cell layer of the hippocampus (*E4bp4^cKO^*) mice were generated by breeding *E4bp4^fl/fl^* mice with mice expressing *Camk2a‐iCre*. Genotyping of mice expressing *Camk2a‐iCre* was performed with primers *Camk2a‐*iCre‐F: 5’‐AGGGTATTGCCTTAGTAGAGGGGCAT‐3’, *Camk2a‐*iCre‐R1: 5’‐GGTGGCAATGGTAGGGTGATCTGAC‐3,’ and *Camk2a‐*iCre‐R2: 5’‐GCACACAGACAGGAGCATCTTC‐3’. Mice were housed in a specific pathogen‐free facility under a 12 h light/dark cycle (unless otherwise specified) with free access to water and food. Male mice (3–4 months) were used for experiments, and experimental procedures were approved by the Institutional Animal Care and Use Committee of Guangzhou University of Chinese Medicine (ZYD‐2020‐145).

### Human Specimens

Whole blood samples were collected from five ICU patients with delirium, five ICU patients without delirium, and five healthy volunteers at different times of the day (i.e., 02:00 AM, 06:00 AM, 10:00 AM, 14:00 PM, 18: 00 PM, and 22:00 PM). Samples were subjected to RNA extraction and qPCR analyses. The study protocol was approved by the Institutional Review Board of the First Affiliated Hospital of Jinan University (KY‐2020‐003). An informed consent was obtained from every participant prior to the study.

### Mouse Models of Delirium

*Drug‐induced delirium*: Knockout (*E4bp4^−/−^*, microglia*^E4bp4‐KO^*, and *E4bp4^cKO^*) mice and controls were treated with LPS (200 µg per kg) plus midazolam (10 mg per kg, i.p., named LM treatment) to induce delirium‐like syndrome as previously described.^[^ ^27^ ^]^ 24 h, 72 h, or 6 days (time points selected based on pilot experiments evaluating the duration of induced delirium) later, mice were subjected to behavioral testing.^[^ ^27^ ^]^ In another set of experiments (for circadian expression profiling), mice were kept under a constant dark condition for 2 days and then treated with LM. 24 h later, mice were sacrificed every 4 circadian hours to collect plasma and hippocampus samples, followed by corticosterone measurement (by using an ELISA kit, Meimian Biotechnology, Jiangsu, China), qPCR, and/or Western blotting analyses.

*Anesthesia/surgery‐induced delirium*: Mice were subjected to a simple laparotomy under anesthesia (named AS treatment) as previously described.^[^ ^28^, ^29^ ^]^ In brief, mice were anesthetized and a longitudinal midline incision was made through the skin, abdominal muscles, and peritoneum from the xiphoid to 0.5 cm proximal to the pubic symphysis. Abdominal organs were partially exposed for 2 min and the incision was sutured with 5‐0 Vicryl thread. 1, 3, 6, or 12 days (time points selected based on pilot experiments evaluating the duration of induced delirium) later, mice were subjected to behavioral testing. In another set of experiments (for circadian expression profiling), mice were treated with AS and then kept under a constant dark condition for 2 days. On the next day, mice were sacrificed every 4 circadian hours to collect plasma and hippocampus samples, followed by corticosterone measurement, qPCR, and/or Western blotting analyses.

### Jet‐Lagged Model

Jet‐lagged mice were established according to a published method.^[^ ^30^ ^]^ In brief, mice were subjected to a jet lag schedule of 8 h light advance of the light/dark cycle every 2 days for 10 days. Control mice were kept under a standard 12 h light/dark cycle. All mice were then transferred to constant darkness. On the next day, mice were subjected to behavioral testing. After another 24 h, mice were sacrificed to collect hippocampus tissues for qPCR analyses.

### Locomotor Activity Analysis

Mice were housed in individual cages equipped with running wheels (Lafayette Instrument, Lafayette, IN) in light‐tight ventilated chambers. After acclimation to the system for 7 days under a 12 h light/dark cycle, mice were treated with LM or AS, and transferred to constant darkness for continuous recording (3–5 days). Data (day 2 after LM or AS treatment) were collected and analyzed using the ClockLab software (Actimetrics, Wilmette, IL).

### EEG (Electroencephalogram) and EMG (Electromyography) Recordings

Mice were anesthetized and mounted in a stereotaxic apparatus. Screw electrodes were inserted into the skull of mice to measure cortical EEG using the following coordinates: +2 mm Bregma, +1 mm midline for first recording electrode; +2 mm Bregma, −1 mm midline for second recording electrode; −2 mm Bregma, −1 mm midline for a reference electrode; and −2 mm Bregma, +1 mm midline for a ground electrode. Stainless steel electrodes were implanted in dorsal neck muscle to measure EMG. After recovery for 6 days, mice (maintained under a 12 h light/dark cycle) were treated with LM. On the next day, mice were subjected to EEG and EMG recordings. In another set of experiments, mice were treated with AS. On day 3, mice were subjected to EEG and EMG recordings. Data were acquired using a tethered data acquisition system with a resolution of 500 Hz (Medusa, Biosignal technologies, Nangjing, China), waveforms visualized using Sirenia Sleep Pro software (Pinnacle technologies, Lawrence, KS). The EEG signals were high‐pass filtered (>0.5 Hz) using a digital filter and EMG was band‐pass filtered between 5 and 45 Hz. Power in the *δ* (0.5–4 Hz), *θ* (5–8 Hz), *α* (9–14 Hz) bands, and *θ* to *δ* band ratio were calculated, and EMG signal was scored in 4 s epochs. All these data were used to define the vigilance states of wake, NREM (non‐rapid eye movement), and REM (rapid eye movement) sleep by an automatic script (Lunion Stage software, LunionData, Nangjing, China).

### SR8278 Treatment

Mice were anesthetized, placed in a stereotaxic frame, and treated with SR8278 or vehicle via microinjections into the hippocampus bilaterally (anteroposterior: −2.9 mm from the bregma, ventral: −3.0 mm below the dura, and lateral: ±3.0 mm from the midline). After 12 h, mice were treated with LM. 24 h later, mice were subjected to behavioral testing.

### Electrophysiology

Hippocampal slices (300 µm thick) were prepared from mouse brain, incubated at 32 °C for 30 min and maintained at 26 °C for 1 h as described previously.^[^ ^64^ ^]^ After recovery, slices were placed in a recording chamber at 25 °C and perfused with oxygenated artificial cerebrospinal fluid (ACSF) containing 126 m m NaCl, 2.5 m m KCl, 1.25 m m NaH_2_PO_4_, 26 m m NaHCO_3_, 10 m m glucose, 2 m m CaCl_2_, and 2 m m MgSO_4_ at a rate of 1 mL min^−1^. Extracellular field EPSPs (fEPSPs) were recorded from the CA1 area using a glass electrode filled with ACSF (2–3 MΩ). Schaffer collateral pathway was stimulated every 30 s using concentric bipolar electrodes. A theta burst stimulation (TBS) protocol (four pulses of 100 Hz repeated three times at 5 Hz, and a 20 s inter‐train interval) was used to induce LTP.^[^ ^64^, ^65^ ^]^ Field potentials were amplified, low‐pass filtered (MultiClamp 700B, Axon Instruments), digitized, and data were analyzed using the Clampex software (Axon Instruments).

### Microglia Depletion

Microglia were depleted by feeding mice a chow diet containing PLX3397 for 14 days as previously described.^[^ ^66^, ^67^ ^]^ Mice were then subjected to LM treatment. After 24 h, behavioral testing was performed and hippocampus samples were collected.

### Behavioral Tests

NOR, Y maze, and OFT tests were performed with mice as previously described^[^ ^68^, ^69^ ^]^ and animal behaviors were recorded by using a SMART video tracking system and analyzed with a SMART 3.0 software (Panlab Harvard Apparatus, Barcelona, Spain). In the NOR test, object preference% = the number of novel or old object exploration/the total number of object exploration. In the Y maze test, spontaneous alternation was calculated as the number of actual alternations divided by the maximum number of alternations.

### Immunofluorescent Staining

Brain samples were fixed, paraffin‐embedded, and cut into 20 µm coronal sections. Sections were blocked with 5% bovine serum albumin in phosphate‐buffered saline (PBS) containing 0.1% Triton X‐100, and then incubated with anti‐Iba1, anti‐GFAP, or anti‐NeuN antibody (Table S3, Supporting Information). After washing with PBS, sections were incubated with a fluorescent secondary antibody and DAPI (4’,6‐diamidino‐2‐phenylindole). Images were captured using a Nikon Optiphot fluorescent microscope (Tokyo, Japan).

### Isolation of Primary Mouse Microglia

Primary mouse microglia were isolated from newborn mice as previously described.^[^ ^32^, ^70^ ^]^ The newborn mice were sacrificed and hippocampus was dissected and incubated in a digestion buffer (Dulbecco's modified Eagle's medium (DMEM) containing 8 u mL^‐1^ papain and 125 u mL^‐1^ DNase) at 37 °C for 20 min. The dissociated cells were collected and resuspended in DMEM with 10% fetal bovine serum (FBS), 1% penicillin/streptomycin, and 5 ng mL^−1^ GM‐CSF. Cells were then seeded into poly‐Dlysine (PDL)‐coated flasks. Flasks were shaken at 200 rpm at 37 °C for 2 h. Cell suspension was centrifuged for 10 min at 200 g and the pellets (microglia) were collected.

### Cell Culture and Treatment

BV2 cells and primary mouse microglia were cultured in DMEM supplemented with 10% FBS at 37 °C in a 5% CO_2_ humidified atmosphere. Cells were transfected with overexpression plasmid or siRNA or control using JetPRIME (Polyplus Transfection, Ill kirch, France). After 24 h, cells were collected for qPCR and Western blotting. In a different set of experiments, transfected cells were stimulated by LPS for 8 h, and cells were collected for qPCR and Western blotting. For inhibition studies, BV2 and primary mouse microglia were pretreated with the selective MEK1/2 inhibitor U0126 (10 µ m ) for 1 h and then treated with LPS or vehicle. After 8 h, cells were collected for qPCR and Western blotting analyses.

### qPCR

Total RNA was extracted with TRIzol reagent following the manufacturer's instructions (Invitrogen, Carlsbad, CA). Total RNA (1 µg) was reversely transcribed into cDNA using PrimeScript RT Master Mix (Takara Bio., Shiga, Japan). qPCR reactions were performed with GoTap qPCR Master Mix (Promega, Madison, WI) using a Biometra Toptical Thermocycler (Analytik Jena, Goettingen, Germany) as previously described.^[^ ^56^ ^]^ Data were normalized to a housekeeping gene (mouse *β‐actin* or human *GAPDH*). Primers are listed in Table S2, Supporting Information.

### Western Blotting

Proteins separated by sodium dodecyl sulfate‐polyacrylamide gel electrophoresis were transferred to polyvinylidene difluoride membranes (Millipore, Bedford, MA). The membrane was blocked in a blocking solution of 2% (w/v) skimmed milk in TBST (0.1% Tween20, 50 m m Tris‐HCl, 140 m m NaCl, and 1 m m MgCl_2_; pH=7.6) for 1 h at room temperature. Then, the membrane was incubated overnight at 4 °C with a primary antibody diluted in the blocking solution. Bands were visualized with enhanced chemiluminescence using an Omega LumG Imaging System (Aplegen, Pleasanton, CA) and band densities were analyzed with FluorChem 5500 (Alpha Innotech, San Leandro, CA). GAPDH, *β*‐tubulin and LaminA/C were used as internal controls. Information of primary antibodies is provided in Table S3, Supporting Information.

### RNA‐Sequencing

RNA was isolated from hippocampus samples using Trizol (Invitrogen, Carlsbad, CA) and quantified using Qubit 2.0 Fluorometer (Life Technologies, Carlsbad, CA). RNA quality was checked using Bioanalyzer 2100 RNA 6000 Nano Kit (Agilent Technologies, Santa Clara, CA), and samples were considered qualified when RIN > 7.7. RNA‐sequencing were performed as described in the authors’ previous reports.^[^ ^50^, ^71^ ^]^ Genes were defined as differentially expressed when *p* < 0.05 and fold change >1.5.

### Single‐Cell RNA‐Sequencing

Four *E4bp4* ^−/−^ mice and four control mice were sacrificed and the hippocampus tissues were isolated. Hippocampus samples of each genotype were pooled. Pooled samples were dissociated using an Adult Brain Dissociation Kit according to the manufacturer's instructions (Miltenyi Biotec, Bergisch Gladbach, Germany). Single‐cell RNA sequencing libraries were prepared using the Chromium Single Cell Controller (10× Genomics, Pleasanton, CA) as previously described.^[^ ^72^ ^]^ Hippocampal cells, diluted appropriately, were mixed with reverse transcription reagent and loaded to a single cell chip, followed by the addition of gel beads and partitioning oil. After generation of emulsion droplet, reverse transcription was performed at 53 °C for 45 min and inactivated at 85 °C for 5 min. cDNA was purified and amplified for 12 cycles on a Bio‐Rad C1000 Touch thermocycler. Amplified cDNA was fragmented, end repaired, A‐tailed and ligated to sequencing adaptor. Final libraries were sequenced on Illumina NovaSeq 6000 (Illumina, San Diego) with a read length of 150 bp. To ensure high‐quality data, only genes expressed in three or more cells, and cells with more than 200 and less than 4500 detected genes, were retained in the dataset. For clustering, signals were cleaned to keep cells with a mitochondrial content of <30% and data normalized to total UMIs and mitochondrial content.

### Luciferase Reporter Assay

BV2 cells were co‐transfected with *Mapk1/Mapk3* luciferase reporter (50 ng), pRL‐nTK (a renilla luciferase reporter, 10 ng), and *E4bp4* overexpression plasmid (or blank pcDNA3.1, 200 ng) using JetPRIME (Polyplus Transfection, Ill kirch, France). 24 h later, cells were collected and luciferase activities were measured using Dual‐Luciferase Reporter Assay System (Promega, Madison, WI). Firefly luciferase activity was normalized to renilla luciferase activity and expressed as a relative luciferase unit (RLU).

### ChIP Assay

ChIP assays were performed using a SimpleChip plus Enzymatic Chromatin IP kit (Cell Signaling Technology, Beverly, MA) as described in the authors’ previous publication.^[^ ^56^ ^]^ Specific primers are provided in Table S1, Supporting Information.

### Statistical Analysis

Data are recorded as mean ± standard errors of the mean (SEM), and outliers were included in data analysis. The amplitudes of rhythmic genes and behaviors were obtained by performing cosinor analysis (https://cosinor.online). Student's *t*‐test (two‐sided) was used to analyze a statistical difference between two groups. One‐way or two‐way ANOVA followed by Bonferroni post hoc test was used for multiple group comparisons. All statistical analyses were performed with GraphPad Prism 8.0 (GraphPad Software Inc., San Diego, CA). The specific tests and number of replicates (*n*) are indicated in the figure legends. The level of significance was set at *p* < 0.05 (*).

## Conflict of Interest

The authors declare no conflict of interest.

## Authors Contribution

*Participated in research design*: MC, KL, ZW, and BW. *Conducted experiments*: MC, LZ, MS, JD, YX, FZ, TZ, and QZ. *Performed data analysis*: MC, LZ, MS, JD, YX, FZ, YL, KL, ZW, and BW. *Wrote or contributed to the writing of the manuscript*: MC and BW.

## Supporting information

Supporting Information

## Data Availability

All data associated with this study are present in the manuscript or the Supporting Information.

## References

[1] K. Gibb , A. Seeley , T. Quinn , N. Siddiqi , S. Shenkin , K. Rockwood , D. Davis , Age Ageing 2020, 49, 352. 32239173 10.1093/ageing/afaa040 PMC7187871

[2] A. Hosie , P. M. Davidson , M. Agar , C. R. Sanderson , J. Phillips , Palliative Med. 2013, 27, 486. 10.1177/0269216312457214 22988044

[3] S. K. Inouye , R. G. Westendorp , J. S Saczynski , Lancet 2014, 383, 911. 23992774 10.1016/S0140-6736(13)60688-1 PMC4120864

[4] E. S. Oh , T. G. Fong , T. T. Hshieh , S. K Inouye , JAMA, J. Am. Med. Assoc. 2017, 318, 1161. 10.1001/jama.2017.12067 PMC5717753 28973626

[5] D. L. Leslie , E. R. Marcantonio , Y. Zhang , L. Leo‐Summers , S. K Inouye , Arch. Intern. Med. 2008, 168, 27. 18195192 10.1001/archinternmed.2007.4 PMC4559525

[6] J. E. Wilson , M. F. Mart , C. Cunningham , Y. Shehabi , T. D. Girard , A. M. J. MacLullich , A. J. C. Slooter , E. E. W. Delirium , Nat. Rev. Dis. Primers 2020, 6, 90. 33184265 10.1038/s41572-020-00223-4 PMC9012267

[7] K. Alagiakrishnan , C. A Wiens , Postgrad. Med. J. 2004, 80, 388. 15254302 10.1136/pgmj.2003.017236 PMC1743055

[8] M. R. Ralph , R. G. Foster , F. C. Davis , M. Menaker , Science 1990, 247, 975. 2305266 10.1126/science.2305266

[9] N. Cermakian , P. Sassone‐Corsi , Nat. Rev. Mol. Cell Biol. 2000, 1, 59. 11413490 10.1038/35036078

[10] D. P. King , J. S Takahashi , Annu. Rev. Neurosci. 2000, 23, 713. 10845079 10.1146/annurev.neuro.23.1.713

[11] S. M. Reppert , D. R Weaver , Nature 2002, 418, 935. 12198538 10.1038/nature00965

[12] N. Gekakis , D. Staknis , H. B. Nguyen , F. C. Davis , L. D. Wilsbacher , D. P. King , J. S. Takahashi , C. J Weitz , Science 1998, 280, 1564. 9616112 10.1126/science.280.5369.1564

[13] K. Kume , M. J. Zylka , S. Sriram , L. P. Shearman , D. R. Weaver , X. Jin , E. S. Maywood , M. H. Hastings , S. M Reppert , Cell 1999, 98, 193. 10428031 10.1016/s0092-8674(00)81014-4

[14] M. K. Bunger , L. D. Wilsbacher , S. M. Moran , C. Clendenin , L. A. Radcliffe , J. B. Hogenesch , M. C. Simon , J. S. Takahashi , C. A Bradfield , Cell 2000, 103, 1009. 11163178 10.1016/s0092-8674(00)00205-1 PMC3779439

[15] D. Dong , D. Yang , L. Lin , S. Wang , B. Wu , Biochem. Pharmacol. 2020, 178, 114045. 32446886 10.1016/j.bcp.2020.114045

[16] I. G. Cowell , H. C Hurst , Nucleic Acids Res. 1994, 22, 59. 8127655 10.1093/nar/22.1.59 PMC307746

[17] T. Ohno , Y. Onishi , N. Ishida , Nucleic Acids Res. 2007, 35, 648. 17182630 10.1093/nar/gkl868 PMC1802629

[18] A. Acharya , V. Rishi , J. Moll , C. Vinson , J. Struct. Biol. 2006, 155, 130. 16725346 10.1016/j.jsb.2006.02.018

[19] S. Kamizono , G. S. Duncan , M. G. Seidel , A. Morimoto , K. Hamada , G. Grosveld , K. Akashi , E. F. Lind , J. P. Haight , P. S. Ohashi , A. T. Look , T. W Mak , J. Exp. Med. 2009, 206, 2977. 19995955 10.1084/jem.20092176 PMC2806474

[20] X. Yu , D. Rollins , K. A. Ruhn , J. J. Stubblefield , C. B. Green , M. Kashiwada , P. B. Rothman , J. S. Takahashi , L. V Hooper , Science 2013, 342, 727. 24202171 10.1126/science.1243884 PMC4165400

[21] M. Keniry , R. K. Dearth , M. Persans , R. Parsons , Discoveries 2014, 2, e15. 26539561 10.15190/d.2014.7 PMC4629104

[22] J. M. Fitzgerald , D. Adamis , P. T. Trzepacz , N. O'Regan , S. Timmons , C. Dunne , D. J Meagher , Med. Hypotheses 2013, 81, 568. 23916192 10.1016/j.mehy.2013.06.032

[23] D. J. Meagher , M. Moran , B. Raju , D. Gibbons , S. Donnelly , J. Saunders , P. T Trzepacz , Psychosomatics 2008, 49, 300. 18621935 10.1176/appi.psy.49.4.300

[24] D. J. Meagher , M. Leonard , S. Donnelly , M. Conroy , D. Adamis , P. T Trzepacz , J. Psychosom. Res. 2012, 72, 236. 22325705 10.1016/j.jpsychores.2011.11.013

[25] T. Sun , Y. Sun , X. Huang , J. Liu , J. Yang , K. Zhang , G. Kong , F. Han , D. Hao , X. Wang , J. Int. Med. Res. 2021, 49, 030006052199050. 10.1177/0300060521990502 PMC7983249 33730927

[26] E. Diaz , I. Diaz , C. Del Busto , D. Escudero , S. Pérez , J Intensive Care Med. 2020, 35, 1497. 31510864 10.1177/0885066619876572

[27] J. Gile , B. Scott , T. Eckle , Crit. Care Med. 2018, 46, e600. 29489460 10.1097/CCM.0000000000003077 PMC5953804

[28] M. Peng , C. Zhang , Y. Dong , Y. Zhang , H. Nakazawa , M. Kaneki , H. Zheng , Y. Shen , E. R. Marcantonio , Z. Xie , Sci. Rep. 2016, 6, 29874. 27435513 10.1038/srep29874 PMC4951688

[29] Y. Zhou , J. Wang , X. Li , K. Li , L. Chen , Z. Zhang , M. Peng , Front. Aging Neurosci. 2020, 12, 582674. 33250764 10.3389/fnagi.2020.582674 PMC7674198

[30] E. Filipski , F. Delaunay , V. M. King , M. W. Wu , B. Claustrat , A. Gréchez‐Cassiau , C. Guettier , M. H. Hastings , L. Francis , Cancer Res. 2004, 64, 7879. 15520194 10.1158/0008-5472.CAN-04-0674

[31] D. Kojetin , Y. Wang , T. M. Kamenecka , T. P Burris , ACS Chem. Biol. 2011, 6, 131. 21043485 10.1021/cb1002575 PMC3042041

[32] P. Griffin , J. M. Dimitry , P. W. Sheehan , B. V. Lananna , C. Guo , M. L. Robinette , M. E. Hayes , M. R. Cedeño , C. J. Nadarajah , L. A. Ezerskiy , M. Colonna , J. Zhang , A. Q. Bauer , T. P. Burris , E. S Musiek , Proc. Natl. Acad. Sci. U.S.A. 2019, 116, 5102. 30792350 10.1073/pnas.1812405116 PMC6421453

[33] E. Colombo , C. Farina , Trends Immunol. 2016, 37, 608. 27443914 10.1016/j.it.2016.06.006

[34] C. A. Hoeffer , W. Tang , H. Wong , A. Santillan , R. J. Patterson , L. A. Martinez , M. V. Tejada‐Simon , R. Paylor , S. L. Hamilton , E. Klann , Neuron 2008, 60, 832. 19081378 10.1016/j.neuron.2008.09.037 PMC2630531

[35] X. L. Wang , S. Kooijman , Y. Gao , L. Tzeplaeff , B. Cosquer , I. Milanova , S. E. C. Wolff , N. Korpel , M. F. Champy , B. Petit‐Demoulière , I. Goncalves Da Cruz , T. Sorg‐Guss , P. C. N. Rensen , J. C. Cassel , A. Kalsbeek , A. L. Boutillier , C. X Yi , Mol. Psychiatry 2021, 26, 6336. 34050326 10.1038/s41380-021-01169-z PMC8760060

[36] S. Balaratnasingam , A. Janca , Pharmacol. Ther. 2012, 134, 116. 22281237 10.1016/j.pharmthera.2012.01.006

[37] S. Cursano , C. R. Battaglia , C. Urrutia‐Ruiz , S. Grabrucker , M. Schön , J. Bockmann , S. Braumüller , P. Radermacher , F. Roselli , M. Huber‐Lang , T. M Boeckers , Mol. Psychiatry 2021, 26, 3778. 32051550 10.1038/s41380-020-0659-y PMC8550963

[38] O. D. Guillamondegui , J. E. Richards , E. W. Ely , J. C. Jackson , K. R. Archer , P. R. Norris , W. T Obremskey , J. Trauma 2011, 70, 910. 21610396 10.1097/TA.0b013e3182114f18

[39] I. Segura , C. Lange , E. Knevels , A. Moskalyuk , R. Pulizzi , G. Eelen , T. Chaze , C. Tudor , C. Boulegue , M. Holt , D. Daelemans , M. Matondo , B. Ghesquière , M. Giugliano , C. Ruiz de Almodovar , M. Dewerchin , P. Carmeliet , Cell Rep. 2016, 14, 2653. 26972007 10.1016/j.celrep.2016.02.047 PMC4805856

[40] S. Yang , C. Gu , E. T. Mandeville , Y. Dong , E. Esposito , Y. Zhang , G. Yang , Y. Shen , X. Fu , E. H. Lo , Z. Xie , Front. Immunol. 2017, 8, 902. 28848542 10.3389/fimmu.2017.00902 PMC5552714

[41] A. Zeisel , A. B. Muñoz‐Manchado , S. Codeluppi , P. Lönnerberg , G. La Manno , A. Juréus , S. Marques , H. Munguba , L. He , C. Betsholtz , C. Rolny , G. Castelo‐Branco , J. Hjerling‐Leffler , S. Linnarsson , Science 2015, 347, 1138. 25700174 10.1126/science.aaa1934

[42] K. Saud , R. Herrera‐Molina , R. Von Bernhardi , Neurotoxic. Res. 2005, 8, 277. 10.1007/BF03033981 16371322

[43] L. Li , D. H. Li , N. Qu , W. M. Wen , W. Q Huang , Cardiology 2010, 117, 207. 21150201 10.1159/000321713

[44] G. W. Jeong , H. H. Lee , W. Lee‐Kwon , H. M Kwon , J. Neuroinflammation 2020, 17, 372. 33292328 10.1186/s12974-020-02007-9 PMC7722447

[45] M. S. Hayden , S. Ghosh , Cell 2008, 132, 344. 18267068 10.1016/j.cell.2008.01.020

[46] H. Lavoie , J. Gagnon , M. Therrien , Nat. Rev. Mol. Cell Biol. 2020, 21, 607. 32576977 10.1038/s41580-020-0255-7

[47] N. Martinez‐Lopez , D. Athonvarangkul , P. Mishall , S. Sahu , R. Singh , Nat. Commun. 2013, 4, 2799. 24240988 10.1038/ncomms3799 PMC3868163

[48] M. Liu , J. Li , P. Dai , F. Zhao , G. Zheng , J. Jing , J. Wang , W. Luo , J. Chen , Stress 2015, 18, 96. 25472821 10.3109/10253890.2014.995085

[49] J. Cerejeira , H. Firmino , A. Vaz‐Serra , E. B Mukaetova‐Ladinska , Acta Neuropathol. 2010, 119, 737. 20309566 10.1007/s00401-010-0674-1

[50] S. Wang , Y. Lin , F. Li , Z. Qin , Z. Zhou , L. Gao , Z. Yang , Z. Wang , B. Wu , Sci. Adv. 2020, 6, eabb5202. 33055157 10.1126/sciadv.abb5202 PMC7556837

[51] K. Yoshida , A. Hashiramoto , T. Okano , T. Yamane , N. Shibanuma , S. Shiozawa , Scand. J. Rheumatol. 2013, 42, 276. 23496259 10.3109/03009742.2013.765031

[52] G. Cavadini , S. Petrzilka , P. Kohler , C. Jud , I. Tobler , T. Birchler , A. Fontana , Proc. Natl. Acad. Sci. U.S.A. 2007, 104, 12843. 17646651 10.1073/pnas.0701466104 PMC1937554

[53] S. R. Allen , H. L Frankel , Surg. Clin. North Am. 2012, 92, 409. 22414419 10.1016/j.suc.2012.01.012

[54] L. K. Fonken , M. G. Frank , M. M. Kitt , R. M. Barrientos , L. R. Watkins , S. F Maier , Brain, Behav., Immun. 2015, 45, 171. 25433170 10.1016/j.bbi.2014.11.009 PMC4386638

[55] P. Griffin , P. W. Sheehan , J. M. Dimitry , C. Guo , M. F. Kanan , J. Lee , J. Zhang , E. S Musiek , eLife 2020, 9, e58765. 33258449 10.7554/eLife.58765 PMC7728439

[56] M. Chen , B. Guan , H. Xu , F. Yu , T. Zhang , B. Wu , Drug Metab. Dispos. 2019, 47, 1333. 31515204 10.1124/dmd.119.088450

[57] B. He , K. Nohara , N. Park , Y. S. Park , B. Guillory , Z. Zhao , J. M. Garcia , N. Koike , C. C. Lee , J. S. Takahashi , S. H. Yoo , Z. Chen , Cell Metab. 2016, 23, 610. 27076076 10.1016/j.cmet.2016.03.007 PMC4832569

[58] Diagnostic and Statistical Manual of Mental Disorders, 5th ed., American Psychiatric Association, Arlington, VA 2013.

[59] D. J. Culley , M. Snayd , M. G. Baxter , Z. Xie , I. H. Lee , J. Rudolph , S. K. Inouye , E. R. Marcantonio , G. Crosby , Front. Aging Neurosci. 2014, 6, 107. 24959140 10.3389/fnagi.2014.00107 PMC4050637

[60] B. Artegiani , A. Lyubimova , M. Muraro , J. H. van Es , A. van Oudenaarden , H. Clevers , Cell Rep. 2017, 21, 3271. 29241552 10.1016/j.celrep.2017.11.050

[61] L. Jiang , Z. Tang , Mol. Med. Rep. 2018, 17, 1499. 29138812 10.3892/mmr.2017.8021 PMC5780090

[62] M. Zhao , T. Zhang , F. Yu , L. Guo , B. Wu , Biochem. Pharmacol. 2018, 152, 293. 29653076 10.1016/j.bcp.2018.04.005

[63] M. Chen , M. Chen , D. Lu , Y. Wang , L. Zhang , Z. Wang , B. Wu , Front. Pharmacol. 2021, 12, 764124. 34887762 10.3389/fphar.2021.764124 PMC8650840

[64] J. E. Choi , S. Kim , J. Lee , K. Kim , B. K Kaang , Exp. Neurobiol. 2018, 27, 344. 30429644 10.5607/en.2018.27.5.344 PMC6221837

[65] A. Kumar , Front. Aging Neurosci. 2011, 3, 7. 21647396 10.3389/fnagi.2011.00007 PMC3102214

[66] M. R. Elmore , A. R. Najafi , M. A. Koike , N. N. Dagher , E. E. Spangenberg , R. A. Rice , M. Kitazawa , B. Matusow , H. Nguyen , B. L. West , K. N Green , Neuron 2014, 82, 380. 24742461 10.1016/j.neuron.2014.02.040 PMC4161285

[67] D. Li , M. Chen , T. Meng , J. Fei , J Neuroinflammation 2020, 17, 109. 32264970 10.1186/s12974-020-01799-0 PMC7140340

[68] X. Yu , A. Zecharia , Z. Zhang , Q. Yang , R. Yustos , P. Jager , A. L. Vyssotski , E. S. Maywood , J. E. Chesham , Y. Ma , S. G. Brickley , M. H. Hastings , N. P. Franks , W. Wisden , Curr. Biol. 2014, 24, 2838. 25454592 10.1016/j.cub.2014.10.019 PMC4252164

[69] D. H. Kim , H. Kwon , J. W. Choi , C. Y. Shin , J. H. Cheong , S. J. Park , J. H Ryu , Prog. Neuro‐Psychopharmacol. Biol. Psychiatry 2020, 102, 109962. 10.1016/j.pnpbp.2020.109962 32428535

[70] S. Du , S. Xiong , X. Du , T. F. Yuan , B. Peng , Y. Rao , J. Vis. Exp. 2021, 10.3791/62237. 33720125

[71] T. Zhang , F. Yu , H. Xu , M. Chen , X. Chen , L. Guo , C. Zhou , Y. Xu , F. Wang , J. Yu , B. Wu , Nat. Commun. 2021, 12, 1216. 33619249 10.1038/s41467-021-21477-w PMC7900242

[72] E. L. Morris , A. P. Patton , J. E. Chesham , A. Crisp , A. Adamson , M. H Hastings , EMBO J. 2021, 40, 108614. 10.15252/embj.2021108614 PMC8521297 34487375

