# Supplementary material for: E4BP4 Coordinates Circadian Control of Cognition in Delirium
Source: Adv Sci (Weinh). 2022 Jun 17;9(23):2200559. doi: 10.1002/advs.202200559 (PMC9376827; doi:10.1002/advs.202200559)
Supplement: Supplementary file 1 — Supporting Information [file ADVS-9-2200559-s001.pdf]

ADVANCED SCIENCE
Open Access

Supporting Information

for *Adv. Sci.*, DOI 10.1002/advs.202200559

E4BP4 Coordinates Circadian Control of Cognition in Delirium

Min Chen, Li Zhang, Mingting Shao, Jianhao Du, Yifei Xiao, Fugui Zhang, Tianpeng Zhang, Yifang Li, Qianqian Zhou, Kaisheng Liu, Zhigang Wang* and Baojian Wu**

Supplementary Data

Manuscript title:

E4BP4 coordinates circadian control of cognition in delirium

Figure S1. (**A**) Experimental protocol used to measure the object preference in the NOR test. (**B**) Representative exploratory paths for three groups of mice in the NOR test. (**C**) Representative exploratory paths for three groups of mice in the Y maze test.

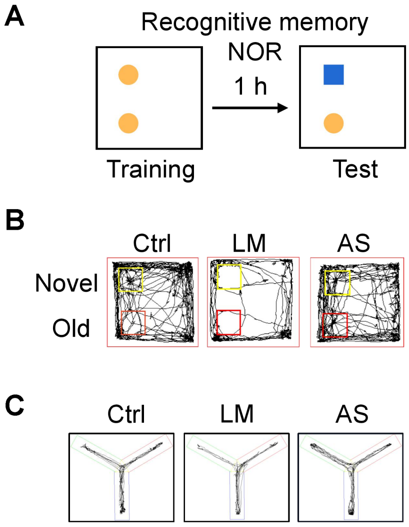

Figure S2. Behavioral deficits recover 6 days after LM treatment and 12 days after AS treatment. (**A**) Object preference for two groups of mice based on the NOR test, which was examined at days 3 and 6 after delirium induction by LM. (**B**) Spontaneous alterations for two groups of mice based on the Y maze test, which were examined at days 3 and 6 after delirium induction by LM. (**C**) Object preference for two groups of mice based on the NOR test, which was examined at days 1, 6 and 12 after delirium induction by AS. (**D**) Spontaneous alterations for two groups of mice based on the Y maze test, which were examined at days 1, 6 and 12 after delirium induction by AS. Data are mean ± SEM (n = 8). *p < 0.05 (t test or two-way ANOVA with Bonferroni post hoc test).

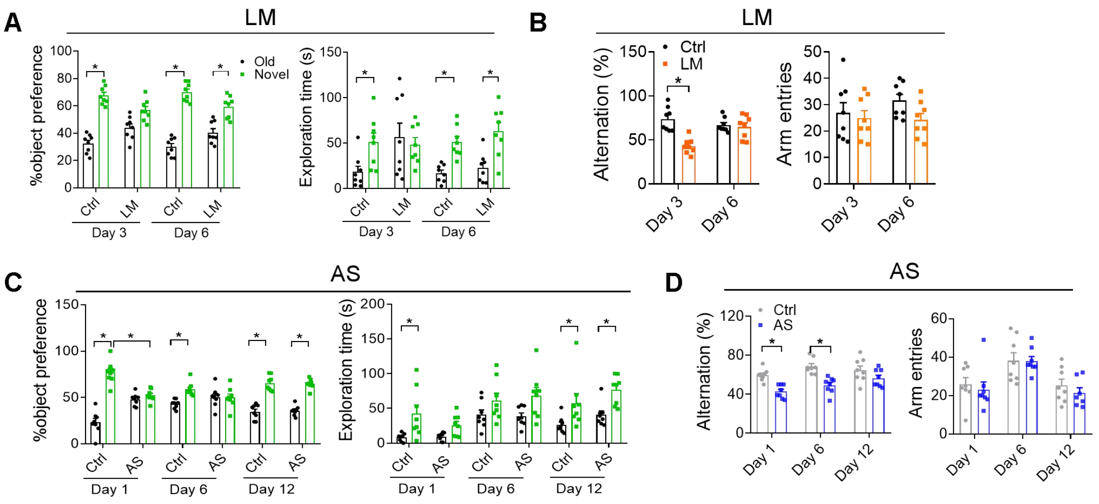

Figure S3. Disruption of circadian rhythms in delirious mice and patients with delirium. (**A**) Representative wheel-running actogram for delirious and control mice. Green arrows indicate the time for LM or AS treatment. Red boxes are actograms of mice after 2 days' treatment. (**B**) Representative traces of EEG and EMG spectra. (**C**) Disrupted circadian pattern of plasma corticosterone in delirious mice (*n* = 4). (**D**) Relative expression of clock genes in the hippocampus from delirious and control mice (*n* = 4). (**E**) Relative expression of clock genes in the whole blood of healthy individuals, delirious and non-delirious ICU patients (*n* = 5). (**F**) Amplitudes of clock genes shown in panel E (*n* = 5). Data are mean ± SEM. *$p < 0.05$ (one-way ANOVA with Bonferroni post hoc test). LD, 12 h light/12 h dark. DD, constant darkness.

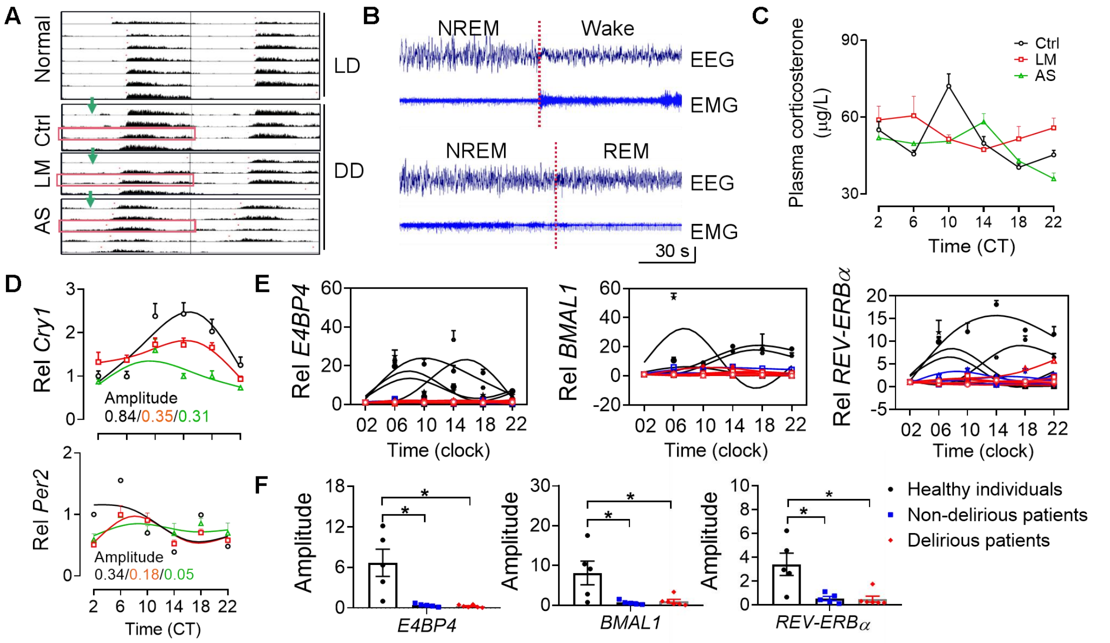

Figure S4. Arm entries for jet-lagged, LL-exposed, *Per2*$^{-/-}$ and control mice based on the Y maze test (*n* = 8 mice). LL, constant lighting.

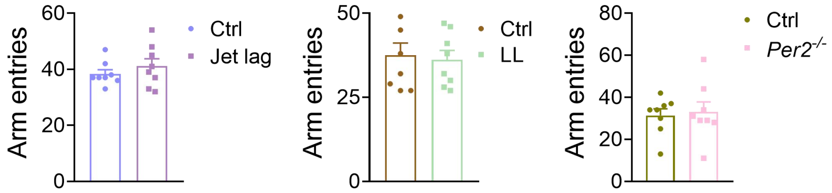

Figure S5. Circadian disruption sensitizes mice to delirium-associated cognitive dysfunction. Object preference for *E4bp4*$^{-/-}$ and control mice based on the NOR test, which was examined at day 3 after delirium induction by AS. Data are mean ± SEM (n = 8). *p < 0.05 (t test or two-way ANOVA with Bonferroni post hoc test).

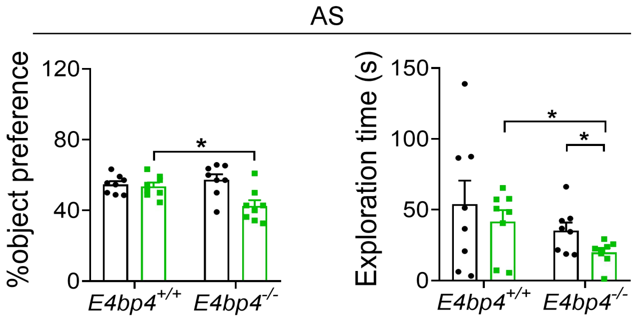

Figure S6. Hippocampal microglial activation underlies delirium-associated cognitive impairment in mice. (**A**) Relative expression of transcripts related to microglial activation in the hippocampus of delirious (induced by AS) and control mice at day 3 (*n* = 4). (**B**) Quantification of $Iba1^+$ cells in the hippocampus of delirious (induced by LM) and control mice (*n* = 3). (**C**) Immunofluorescent staining of hippocampus from delirious (induced by AS) and control mice using the microglial marker Iba1 (green) and DAPI (blue). Scale bar, 20 μm. (**D**) Relative expression of astrocyte marker transcripts in the hippocampus of delirious (induced by LM) and control mice at day 1 (*n* = 4). (**E**) Immunofluorescent staining of hippocampus from three groups of mice using the astrocyte marker GFAP. Scale bar, 100 μm. (**F**) Quantification of $GFAP^+$ cells in the hippocampus of delirious and control mice (*n* = 3). (**G**) Quantification of $Iba1^+$ cells in the hippocampus of PLX3397-treated and control mice after delirium induction by LM (*n* = 3). (**H**) mRNA levels of inflammatory factors in PLX3397-treated mice after delirium induction by LM (*n* = 4). (**I**) Protein levels of IL-1β and TNFα in PLX3397-treated mice after delirium induction by LM. Data are mean ± SEM. *$p < 0.05$ (t test).

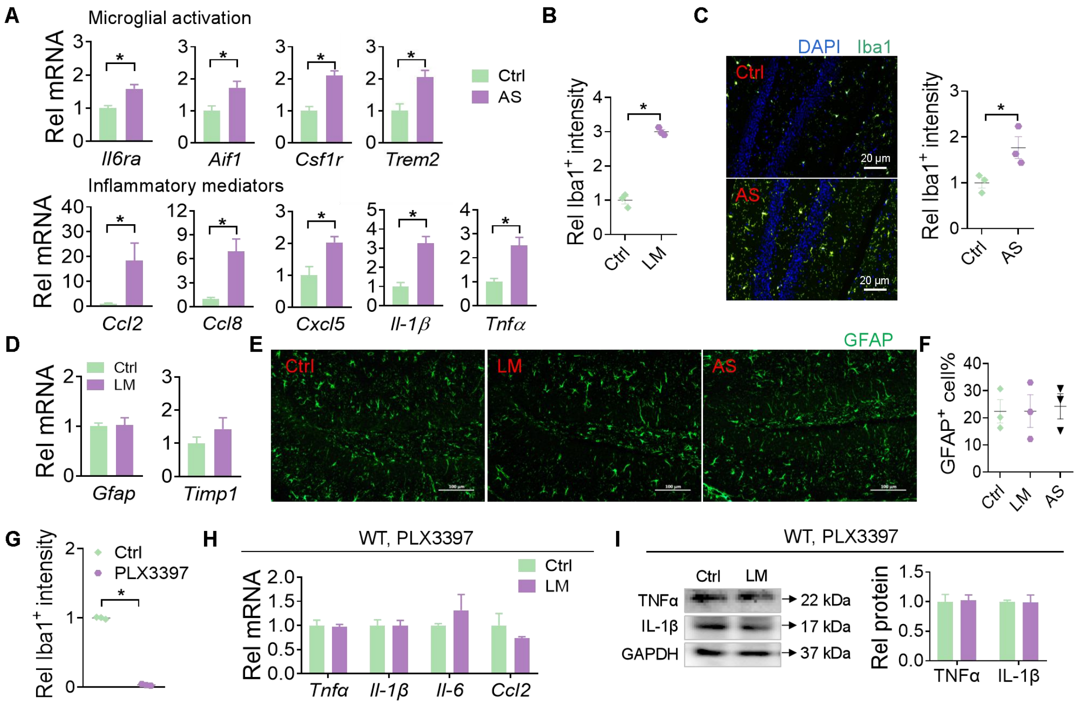

Figure S7. Relative expression of transcripts related to microglial activation in the hippocampus of *E4bp4*$^{-/-}$ and control mice at day 3 after AS treatment.

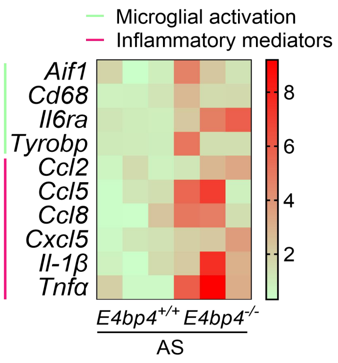

Figure S8. (**A**) mRNA levels of inflammatory factors in PLX3397-treated *E4bp4*$^{-/-}$ mice at day 1 after delirium induction by LM (*n* = 4). (**B**) Protein levels of IL-1β and TNFα in PLX3397-treated *E4bp4*$^{-/-}$ mice at day 1 after delirium induction by LM (*n* = 4). Data are mean ± SEM.

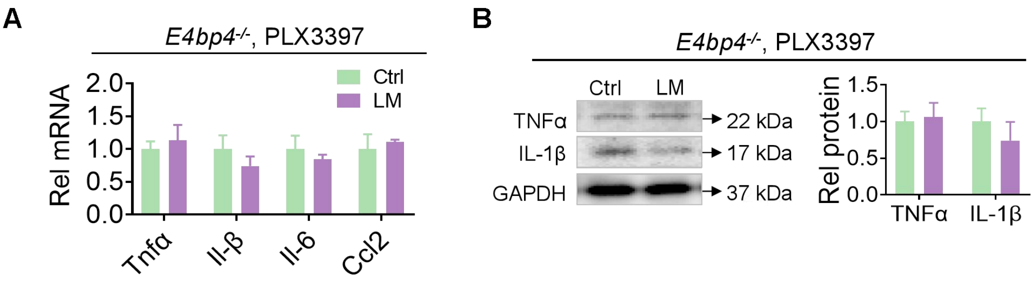

Figure S9. Behavioral tests of *E4bp4^{cKO}* mice. (**A**) Object preference for *E4bp4^{cKO}* and control mice based on the NOR test, which was examined before (day 0) and at days 1 & 6 after delirium induction by LM. (**B**) Spontaneous alterations for *E4bp4^{cKO}* and control mice based on the Y maze test, which were examined before (day 0) and at days 1 & 6 after delirium induction by LM. Data are mean ± SEM (n = 8). *p < 0.05 (t test).

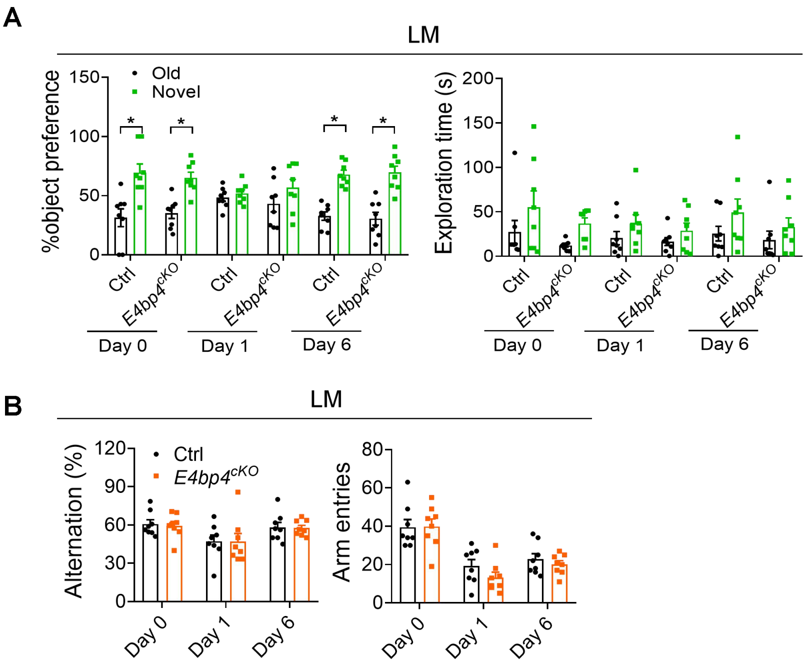

Figure S10. (**A**) Immunofluorescent staining of the neuronal marker NeuN (green) in the hippocampus from *E4bp4*$^{-/-}$ and control mice at day 1 after LM treatment. Scale bar, 100 μm. The right panel shows the quantification of the NeuN^{+} cells (n = 3). (**B**) Immunofluorescent staining of the astrocyte marker GFAP (green) in the hippocampus from *E4bp4*$^{-/-}$ and control mice at day 1 after LM treatment. Scale bar, 100 μm. The right panel shows the quantification of the GFAP^{+} cells (n = 3). (**C**) Relative expression of *Bdnf*, *TrkB, Tjp1* and *Ocln* mRNAs in *E4bp4*$^{-/-}$ and control mice at day 1 after LM treatment (n = 4). (**D**) Protein levels of BDNF, ZO-1 and Occludin in *E4bp4*$^{-/-}$ and control mice at day 1 after LM treatment (n = 4). (**E**) Relative expression of *Hif1α, Vegfa* and *Phd2* mRNAs in *E4bp4*$^{-/-}$ and control mice at day 1 after LM treatment (n = 4). Data are mean ± SEM.

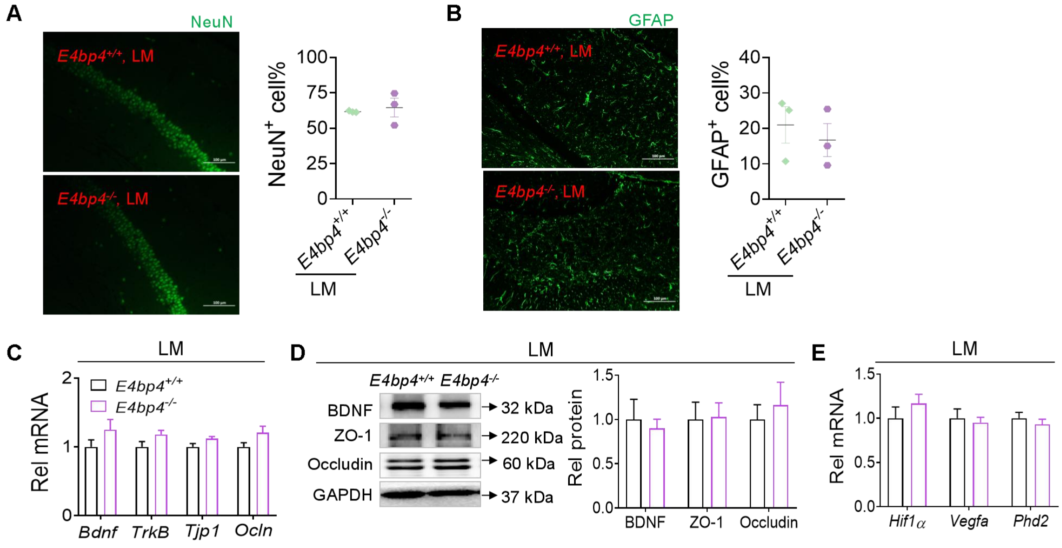

Figure S11. Single-cell RNA-sequencing metrics and quality control plots. (**A**) Number of UMIs detected in each library. (**B**) Number of genes detected in each library. (**C**) Percent ribosome detected in each library. (**D**) Percent mitochondria in each library. (**E**) Expression of known marker genes for major cell types. (**F**) Cell distribution for each cell type.

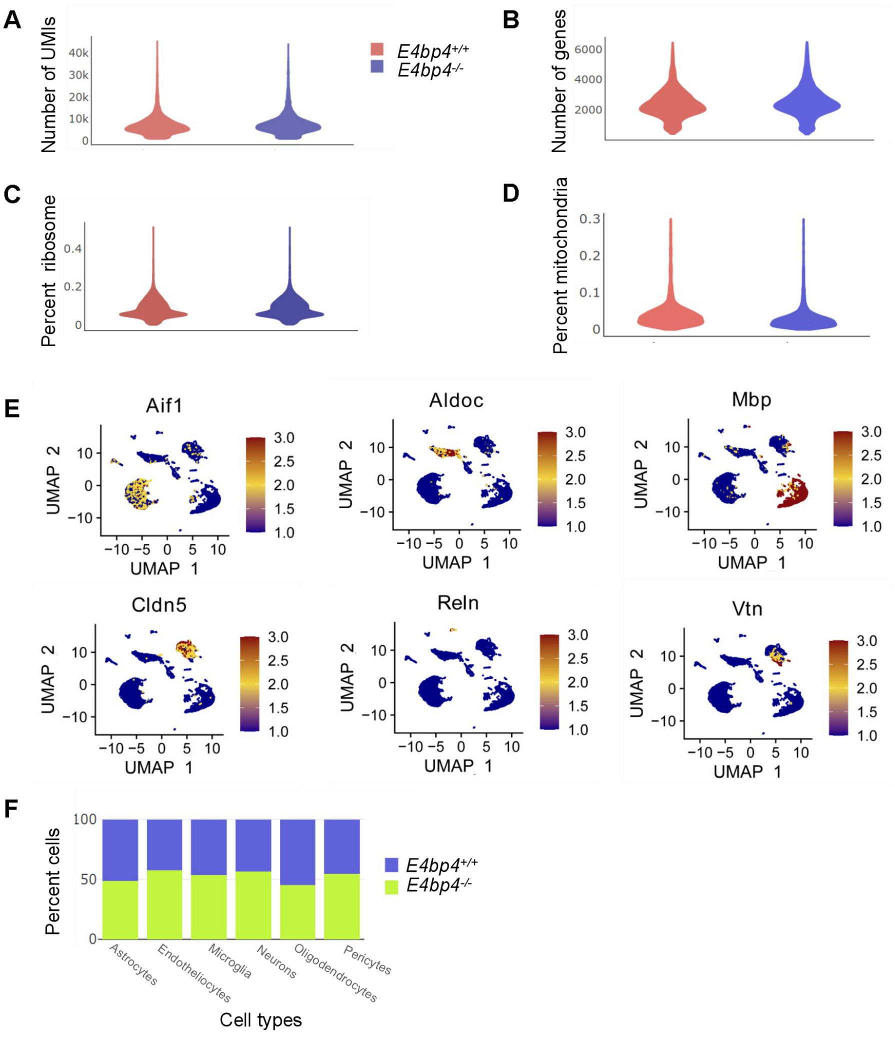

Figure S12. *E4bp4* deficiency promotes microglial activation in mouse hippocampus. (**A**) Relative expression of transcripts related to microglial activation in the hippocampus of *E4bp4*$^{-/-}$ and control mice (n = 4). (**B**) Immunofluorescent staining of hippocampus from *E4bp4*$^{-/-}$ and control mice. Scale bar, 20 μm. (**C**) Quantification of Iba1^{+} cells in the hippocampus of mice (n = 3). Data are mean ± SEM. *p < 0.05 (t test).

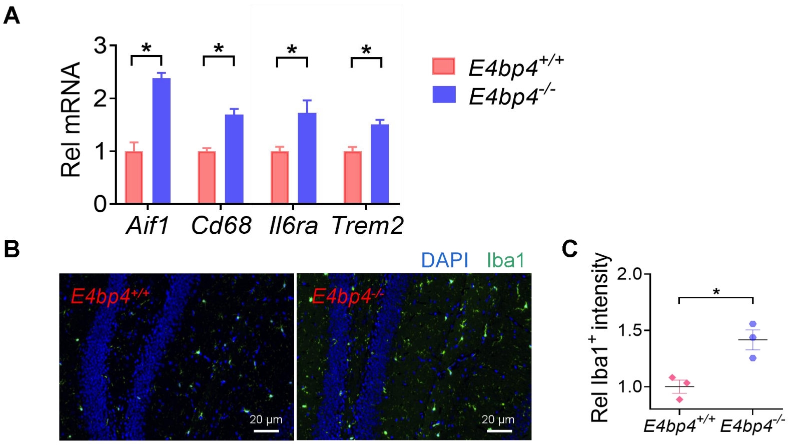

Figure S13. Effects of *E4bp4* loss on total p-MEK protein in the hippocampus of mice at day 1 after LM treatment and at day 3 after AS treatment (**A**), on autophagy proteins ATG7 and LC3 in the hippocampus of mice at day 1 after LM treatment (**B**), and on the expression of genes involved in ERK1/2 cascade in the hippocampus of mice at day 1 after LM treatment (**C**). Data are mean ± SEM (n = 4). *p < 0.05 (t test).

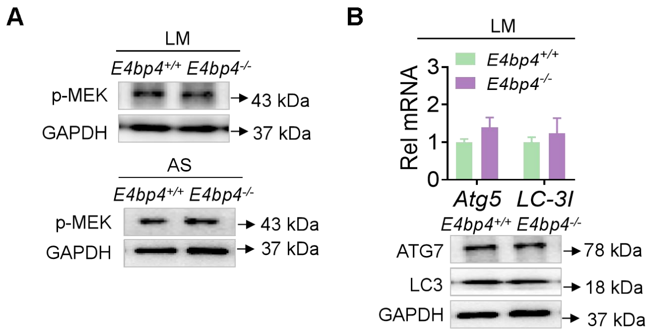

Figure S14. Behavioral characterization of *E4bp4$^{-/-}$* mice. (**A**) Representative wheel-running actogram for *E4bp4$^{-/-}$* and control mice. (**B**) Daily and circadian wheel-running activities recorded at a 1-min interval for *E4bp4$^{-/-}$* and control mice. (**C**) Daily patterns of wake, NREM and REM sleep time for *E4bp4$^{-/-}$* and control mice (*n* = 4). Data are mean ± SEM (*n* = 4). LD, 12 h light/12 h dark. DD, constant darkness.

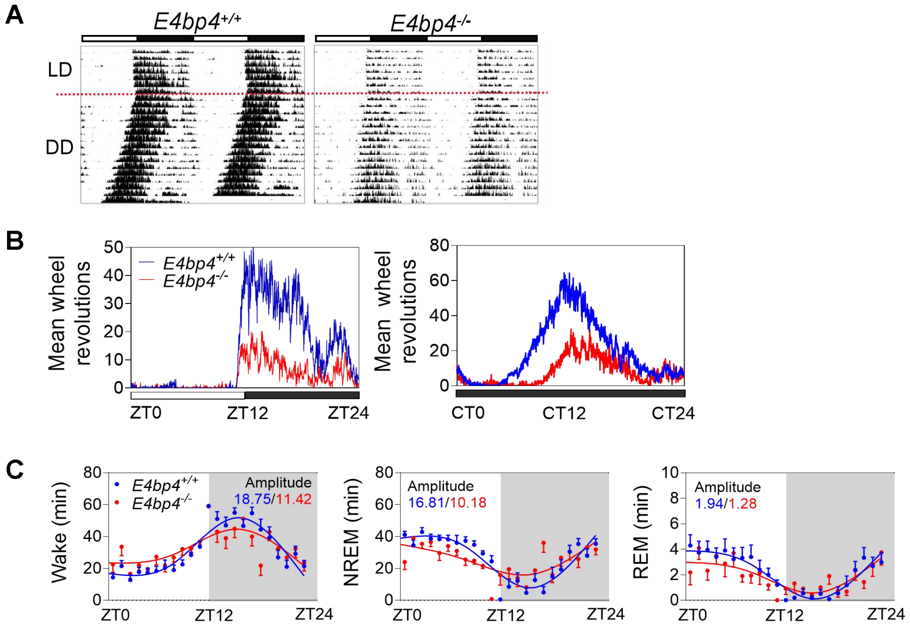

Figure S15. Circadian mRNAs of *Mapk1* and *Mapk3* in mouse hippocampus. Data are mean ± SEM (*n* = 4).

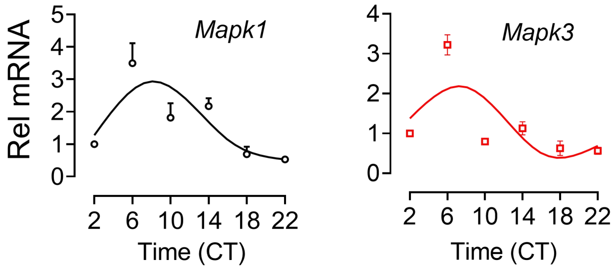

Supplementary table 1. Oligonucleotides used in this study.

	**Forward (5'-3' Sequence)**	**Reverse (5'-3' Sequence)**
siRNA		
siE4bp4	GCACAAGCUUCGGAUUAAATT	UUUAAUCCGAAGCUUGUGCTT
Control	UUCUCCGAACGUGUCACGUTT	ACGUGACACGUUCGGAGAATT
ChIP		
Mapk1-E4bp4	CTCACAAGTGGTGGGAATC	TGGGTAGGGACACTGGAA
Mapk3-E4bp4	CTATGATGGCATCCAATG	AAACAACCAGAACCCTGT

Supplementary table 2. Primer sequences for qPCR.

Gene	Forward (5'-3' Sequence)	Reverse (5'-3' Sequence)
mBmal1	CTCCAGGAGGCAAGAAGATTC	ATAGTCCAGTGGAAGGAATG
mE4bp4	TATTGGGAGAAACGGCGGAAA	AGCCTTGGATGTCTGGTAGTC
mCry1	CCCAGGCTTTTCAAGGAATGGAAC	GCAGGGAGTTTGCATTCATTCGAG
mPer2	CCACACTTGCCTCCGAAATA	ACTGCCTCTGGACTGGAAGA
mRev-erbα	TTTTTCGCCGGAGCATCCAA	ATCTCGGCAAGCATCCGTTG
mAif1	CTTGAAGCGAATGCTGGAGAA	GGCAGCTCGGAGATAGCTTT
mCsf1r	TGTCATCGAGCCTAGTGGC	CGGGAGATTCAGGGTCCAAG
mCd68	TGTCTGATCTTGCTAGGACCG	GAGAGTAACGGCCTTTTTGTGA
mIl6ra	GCCACCGTTACCCTGATTTG	TCCTGTGGTAGTCCATTCTCTG
mTyrobp	CCCAAGATGCGACTGTTCTTC	GTCCCTTGACCTCGGGAGA
mTrem2	CTGGAACCGTCACCATCACTC	CGAAACTCGATGACTCCTCGG
mCcl2	CCACAACCACCTCAAGCACT	AGGCATCACAGTCCGAGTCA
mCcl5	GCTGCTTTGCCTACCTCTCC	TCGAGTGACAAACACGACTGC
mCcl8	TCTACGCAGTGCTTCTTTGCC	AAGGGGGATCTTCAGCTTTAGTA
mCxcl5	TGCCCTACGGTGGAAGTCATA	TGCATTCCGCTTAGCTTTCTTT
mIl-1β	GGCTGTATTCCCCTCCATCG	AAGGTGCTGATCTGGGTTGG
mIl-6	TAGTCCTTCCTACCCCAATTTCC	TTGGTCCTTAGCCACTCCTTC
mTnfα	AGGGTCTGGGCCATAGAACT	CCACCACGCTCTTCTGTCTAC
mGfap	CCCTGGCTCGTGTGGATTT	GACCGATACCACTCCTCTGTC
mTimp1	CGAGACCACCTTATACCAGCG	ATGACTGGGGTGTAGGCGTA
mMapk1	TTGCTTTCTCTCCCGCACAAA	AGAGCCTGTTCAACTTCAATCC
mMapk3	TCCGCCATGAGAATGTTATAGGC	GGTGGTGTTGATAAGCAGATTGG
mMap2k1	GAGTGCAACTCCCCGTACATC	TTCTCCCGAAGATAGGTCAGG
mTlr4	GCCTTTCAGGGAATTAAGCTCC	GATCAACCGATGGACGTGTAAA
mBraf	TGATGCGCTGTCTTCGGAAAT	GCCAGGCTCAAAATCAAACACT
mRaf	GATGGCAAGCTCACGGATTCT	TGTAAGCTCATTCCATTCCGC
mRem1	GAGGTGTGTACGAGAGAACGC	GGCACCAGCTTTCATCCAGTT
mKras	CAAGAGCGCCTTGACGATACA	CCAAGAGACAGGTTTCTCCATC
mHras1	TTTGTGGACGAGTATGATCCCA	TGCTCCCTGTACTGATGGATG
mBdnf	TTACCTGGATGCCGCAAACAT	TGACCCACTCGCTAATACTGTC
mTrkB	CCGCTAGGATTTGGTGTACTG	CCGGGTCAACGCTGTTAGG
mTjp1	GAGCGGGCTACCTTACTGAAC	GTCATCTCTTTCCGAGGCATTAG
mOcln	TTGAAAGTCCACCTCCTTACAGA	CCGGATAAAAAGAGTACGCTGG
mHif1α	ACCTTCATCGGAAACTCCAAAG	CTGTTAGGCTGGGAAAAGTTAGG
mVegfa	CTGCCGTCCGATTGAGACC	CCCCTCCTTGTACCACTGTC
mPhd2	AGTCCTTGGAGTCTAGCCGAG	TGGCAGTGGTCGTAGTAGCA
mAtg7	GCGAAGGTCAGGAGCAGAA	GCGAAGGTCAGGAGCAGAA
mPpib	TCCACACCCTTTTCCGGTCC	CAAAAGGAAGACGACGGAGC
mβ-actin	GGCTGTATTCCCCTCCATCG	CCAGTTGGTAACAATGCCATGT

**Gene**	**Forward (5'-3' Sequence)**	**Reverse (5'-3' Sequence)**
hBMAL1	TTAAGAGGTGCCACCAATCC	TTCCCTCGGTCACATCCTAC
hE4BP4	AGGGAAGCTGCAGAAGTCCTGAAA	AGTTGCTGGAGGATCGGTTGACTT
hREV-ERBα	CCAACAACAACACAGGTGGCG	GGGGATGGTGGGAAGTAGGT
hGAPDH	CATGAGAAGTATGACAACAGCCT	AGTCCTTCCACGATACCAAAGT

M, mouse; h, human.

Supplementary table 3. Antibodies used for Western blotting and immunofluorescent staining in this study.

Primary or secondary	Antibody	Species	Dilution	Source	Cat No.	Application
1st	E4BP4	rabbit	1:3000	Proteintech	11773-1-AP	WB
1st	p-ERK1/2	rabbit	1:5000	Proteintech	28733-1-AP	WB
1st	ERK1/2	rabbit	1:1000	Proteintech	51068-1-AP	WB
1st	p-p65	rabbit	1:1000	CST	#3033	WB
1st	p65	rabbit	1:1000	CST	D14E12	WB
1st	IL-1β	goat	1:1000	R&D Systems	AF-401-NA	WB
1st	TNF-α	mouse	1:4000	Proteintech	60291-lg	WB
1st	Iba1	rabbit	1:1000	Abcam	ab178847	WB
1st	GFAP	rabbit	1:5000	Proteintech	16825-1-AP	WB
1st	p-MEK1	mouse	1:5000	Proteintech	67873-1-lg	WB
1st	ZO-1	rabbit	1:1000	Proteintech	21773-1-AP	WB
1st	Occludin	rabbit	1:1000	Abcam	ab222691	WB
1st	BDNF	mouse	1:2000	Proteintech	66292-1-lg	WB
1st	LC3	rabbit	1:2000	Proteintech	14600-1-AP	WB
1st	ATG7	rabbit	1:1000	Proteintech	10088-2-AP	WB
1st	GAPDH	rabbit	1:2000	Abcam	ab9485	WB
1st	β-tubulin	rabbit	1:2000	Proteintech	10094-1-AP	WB
1st	LaminA/C	rabbit	1:2000	Proteintech	10298-1-AP	WB
1st	GFAP	rabbit	1:500	ServiceBio	GB11096	IF
1st	NeuN	mouse	1:500	Abcam	ab104224	IF
1st	Iba1	rabbit	1:200	Abways	CY7217	IF
2nd	goat anti-rabbit IgG-HRP	rabbit	1:5000	HuaBio	HA1001	WB
2nd	goat anti-mouse IgG-HRP	mouse	1:3000	HuaBio	HA1006	WB
2nd	FITC-labeled Goat Anti-Rabbit IgG (H+L)	rabbit	1:300	ServiceBio	GB22303	IF
2nd	FITC-labeled Goat Anti-Mouse IgG (H+L)	mouse	1:200	Thermo Fischer	GB22301	IF

IF, immunofluorescent staining.
